# Pharmacological and psychotherapeutic interventions for management of obsessive-compulsive disorder in adults: a systematic review and network meta-analysis

**DOI:** 10.1016/S2215-0366(16)30069-4

**Published:** 2016-08

**Authors:** Petros Skapinakis, Deborah M Caldwell, William Hollingworth, Peter Bryden, Naomi A Fineberg, Paul Salkovskis, Nicky J Welton, Helen Baxter, David Kessler, Rachel Churchill, Glyn Lewis

**Affiliations:** a Division of Psychiatry, University College London, London, UK; b Department of Psychiatry, University of Ioannina School of Medicine, University of Ioannina, Ioannina, Greece; c School of Social and Community Medicine, University of Bristol, Bristol, UK; d Department of Postgraduate Medicine, University of Hertfordshire, Hatfield, UK; e Highly Specialised Obsessive-Compulsive Disorder and Body Dysmorphic Disorder Services, Hertfordshire Partnership University NHS Foundation Trust, Hertfordshire, UK; f Department of Psychology, University of Bath, Bath, UK; g Centre for Reviews and Dissemination, University of York, York, UK

## Abstract

**Background:**

Several interventions are available for management of obsessive-compulsive disorder in adults, but few studies have compared their relative efficacy in a single analysis. We aimed to simultaneously compare all available treatments using both direct and indirect data.

**Methods:**

In this systematic review and network meta-analysis, we searched the two controlled trials registers maintained by the Cochrane Collaboration Common Mental Disorders group for trials published up to Feb 16, 2016. We selected randomised controlled trials in which an active psychotherapeutic or pharmacological intervention had been used in adults with obsessive-compulsive disorder. We allowed all comorbidities except for schizophrenia or bipolar disorder. We excluded studies that focused exclusively on treatment-resistant patient populations defined within the same study. We extracted data from published reports. The primary outcome was symptom severity as measured by the Yale-Brown Obsessive Compulsive Scale. We report mean differences with 95% credible intervals compared with placebo. This study is registered with PROSPERO, number CRD42012002441.

**Findings:**

We identified 1480 articles in our search and included 53 articles (54 trials; 6652 participants) in the network meta-analysis. Behavioural therapy (mean difference −14·48 [95% credible interval −18·61 to −10·23]; 11 trials and 287 patients), cognitive therapy (−13·36 [–18·40 to −8·21]; six trials and 172 patients), behavioural therapy and clomipramine (−12·97 [–19·18 to −6·74]; one trial and 31 patients), cognitive behavioural therapy and fluvoxamine (−7·50 [–13·89 to −1·17]; one trial and six patients), cognitive behavioural therapy (−5·37 [–9·10 to −1·63]; nine trials and 231 patients), clomipramine (−4·72 [–6·85 to −2·60]; 13 trials and 831 patients), and all SSRIs (class effect −3·49 [95% credible interval −5·12 to −1·81]; 37 trials and 3158 patients) had greater effects than did drug placebo. Clomipramine was not better than were SSRIs (−1·23 [–3·41 to 0·94]). Psychotherapeutic interventions had a greater effect than did medications, but a serious limitation was that most psychotherapeutic trials included patients who were taking stable doses of antidepressants (12 [80%] of the 15 psychotherapy trials explicitly allowed antidepressants).

**Interpretation:**

A range of interventions is effective in the management of obsessive-compulsive disorder, but considerable uncertainty and limitations exist regarding their relative efficacy. Taking all the evidence into account, the combination of psychotherapeutic and psychopharmacological interventions is likely to be more effective than are psychotherapeutic interventions alone, at least in severe obsessive-compulsive disorder.

**Funding:**

National Institute for Health Research.

## Introduction

Obsessive compulsive disorder is considered the fourth most common mental disorder in high-income countries and ranks as the tenth leading cause of disability worldwide.^1^, ^2^ It is associated with increased mortality^3^ and can have a substantial impact on quality of life for both patients and family members or carers.^2^ Clomipramine and the SSRIs are currently recommended for pharmacological management of the disease.^4^ Psychotherapies and especially behavioural or cognitive behavioural interventions have been developed^5^, ^6^ and are also recommended.^7^

Previous systematic reviews and meta-analyses have generally compared the efficacy of pharmacological interventions with placebo, not with each other.^8^, ^9^, ^10^ Psychotherapeutic interventions have typically been compared with a waiting list or other inactive therapy.^7^, ^11^ Only a few studies have directly compared psychotherapeutic with pharmacological interventions or combinations of them, and their results are inconclusive.^7^ In the absence of available head-to-head comparisons, indirect evidence can be used to enhance the existing evidence base. Indirect comparisons between different medications have been done in the past, but statistical methods appropriate for such comparisons were poorly developed at that time.^10^ Network meta-analysis is a method of synthesising information from a network of trials addressing the same question, but involving different interventions. It aims to combine direct and indirect evidence into a single effect size and rank all available treatments in terms of efficacy, providing estimates for interventions even if they have not been directly compared. This approach has been applied successfully to schizophrenia, bipolar disorder, depression, and certain anxiety disorders (social phobia and generalised anxiety disorder), but not obsessive-compulsive disorder. We therefore did a systematic review and network meta-analysis with the aim to simultaneously compare all available treatments using both direct and indirect data.^12^ A more detailed report than this one will be published, and data collected for children and adolescents will also be separately published.

**Research in context**

**Evidence before this study**

During the protocol stage of our project (May 1 to June 30, 2013), we did a scoping search of the literature. We used the two specialised registers of controlled trials maintained and administered by the Cochrane Collaboration Common Mental Disorders Group. We searched the registers using the generic term “condition = obsess* OR compulsi*”, with no language or date restrictions. We found that the latest comprehensive review had been published in 2006 and specific meta-analyses had been published in 2008. Since then, several new trials have been done. Previous systematic reviews and meta-analyses have generally focused on the comparison between antidepressant medications and placebo or psychotherapeutic interventions and a waiting list. Few studies have directly compared the relative efficacy of serotonergic antidepressants versus each other, behavioural-type psychotherapies versus each other, or medications versus psychotherapies. Clinicians are often interested in pragmatic comparisons (Are all SSRIs equally effective? Is clomipramine more effective than are SSRIs? Is cognitive behavioural psychotherapy more effective than are medications?), but these questions have been examined in few studies in the past using statistical methods that have not always taken into account the complexity of such comparisons. We therefore did a network meta-analysis with the aim to simultaneously compare in a single analysis and rank in terms of efficacy all available interventions for management of obsessive-compulsive disorder in adults.

**Added value of this study**

We found small differences in efficacy between medications, and the hypothesis of clomipramine being better than SSRIs was not confirmed. Although certain psychotherapies were associated with larger effects than were medications, we underline an important limitation that, in most psychotherapeutic trials, patients who were taking stable doses of antidepressants were not excluded and therefore these therapies cannot be considered as pure monotherapies.

**Implications of all the available evidence**

Taking all evidence into account, the combination of psychotherapies with medications is possibly the most effective intervention and clinicians should consider this option more often than at present for patients with severe obsessive-compulsive disorder. Psychotherapy is effective in symptomatic patients taking antidepressant medications, and its effect as monotherapy is not known. Future research should try to differentiate more clearly the effect of medications versus psychotherapy and monotherapy versus combined therapy, avoiding the limitations that we have underlined in this study.

## Methods

### Search strategy and selection criteria

In this systematic review and network meta-analysis, we searched the two controlled trials registers maintained by the Cochrane Collaboration Common Mental Disorders group for trials published up to Feb 16, 2016, by experienced staff of the Cochrane Common Mental Disorders group using their standard methodology. Reports of trials for inclusion in the Group's registers are collated from routine (weekly), generic searches of MEDLINE, Embase, and PsycINFO; quarterly searches of the Cochrane Central Register of Controlled Trials; and review-specific searches of additional databases. We searched the registers using the generic term “condition = obsess* OR compulsi*”, with no language restrictions. We included studies in the review if they were randomised controlled trials of adult patients with a diagnosis of obsessive-compulsive disorder. We allowed all comorbidities except for schizophrenia or bipolar disorder. We excluded studies that focused exclusively on treatment-resistant patient populations defined within the same study.

Eligible experimental interventions were all antidepressants^7^ and psychotherapeutic interventions^7^ recommended by current guidelines—ie, behavioural therapy, including exposure and response prevention but not explicit cognitive techniques (such as cognitive restructuring); cognitive therapy, including cognitive restructuring but not explicit behavioural techniques; and cognitive behavioural therapy (CBT). In psychotherapy trials that used both an individual and group format, we extracted data only for groups with the individual format. Eligible control interventions were drug placebo, psychological placebo (any credible psychological intervention that includes only non-specific components of therapy, such as general stress management or relaxation), and any other non-specific psychotherapeutic relationship. Inclusion and exclusion criteria were independently assessed by two reviewers (HB and PSk) and validated by one reviewer (PSk). For studies that were excluded, we noted the main reason for exclusion.

### Data analysis

Data extraction was done independently by two reviewers (HB and PSk) and validated by one reviewer (PSk). We used standardised data extraction Word forms and structured Excel spreadsheets to extract data from published reports. In cases of duplicate data, we selected the manuscript with the largest sample. We also considered preliminary congress abstracts duplicate and did not select them if a full article had been published after the congress. We extracted data for inclusion and exclusion criteria (study design, experimental intervention, control intervention, age range, primary diagnosis, comorbid diagnoses, and use of diagnostic criteria), general details of the study (country, treatment setting, and length of follow-up), details of continuous outcome assessment (number of patients eligible for randomisation, randomised, dropped out, and remaining at the end of study, and baseline, end of treatment, and change from baseline Yale-Brown Obsessive Compulsive Scale [YBOCS] scores, with SDs), and details of the risk of bias assessment (intention-to-treat analysis, use of methods for handling missing data, and dropouts).

For the quantitative synthesis, the primary outcome measure was continuous and it was symptom severity as measured by YBOCS.^13^ Our preferred measure was mean change from baseline score. For studies in which this measure was not reported, we used mean YBOCS scores at the end of study after checking that YBOCS at baseline was balanced across groups. We report mean differences with 95% credible intervals compared with placebo. We assessed risk of bias using the criteria suggested by the Cochrane Collaboration Handbook.^14^ We included studies with a high risk of bias in the main analysis but did sensitivity analyses to examine the effect of excluding them.

We did pairwise and network meta-analyses for efficacy. We excluded studies that did not use YBOCS. This post-hoc decision was made for two reasons: YBOCS is the only available clinician-rated scale that has been extensively validated in controlled trials worldwide^13^ and use of a single scale allowed us to use the mean difference instead of the standardised mean difference, avoiding the methodological and interpretational difficulties associated with use of standardised mean difference.^14^ Where possible, we derived missing SDs from reported statistics following guidance in the Cochrane Collaboration Handbook.^14^ Where possible, we analysed the intention-to-treat population; otherwise, we used reported results for participants who completed the study.

We did all analyses in a Bayesian framework using OpenBUGS version 3.2.3. We used the random-effect models described by Dias and colleagues,^15^ modified to incorporate an additional class hierarchy,^16^ such that all SSRIs were assumed to be similar, with a common class mean effect and between-SSRI variability about this class mean. We used flat priors for all parameters. We assessed heterogeneity by examining the posterior median of the between-study heterogeneity parameter from the random-effects model. To assess variability within studies, we used what was reported by trial authors. For continuous measures SDs were reported and for ratio measures typically SEs. However, where these statistics were not reported, we used methods recommended by the Cochrane Collaboration Handbook^14^ (eg, estimation of SEs from CIs). We measured goodness of fit with the posterior mean of the residual deviance. To assess inconsistency between direct and indirect evidence, we compared the fit of a model assuming consistency with that of one that relaxes this assumption (unrelated mean-effects model).^17^ We also compared the results of the pairwise meta-analysis with those of the network meta-analysis. All OpenBUGS code is available in the appendix.

Preplanned sensitivity analyses excluded studies at high risk of bias for the following domains: masking of the outcome assessor, incomplete outcome data, and high overall attrition or evidence of differential attrition between groups. We present the results both before (ie, the full dataset) and after excluding waiting list controlled trials. These trials are non-masked and evidence exists that they lead to biased results in favour of the active psychotherapeutic interventions.^18^, ^19^, ^20^ We did separate meta-regressions assuming a common interaction term for the following study-level characteristics: length of trial, publication date, industry sponsorship, and inclusion of patients with current comorbid depression. This study is registered with PROSPERO, number CRD42012002441.

### Role of the funding source

The funder had no role in study design, data collection, data analysis, data interpretation, or writing of the report. The corresponding author had full access to all the data in the study and had final responsibility for the decision to submit for publication.

## Results

**Figure 1:**
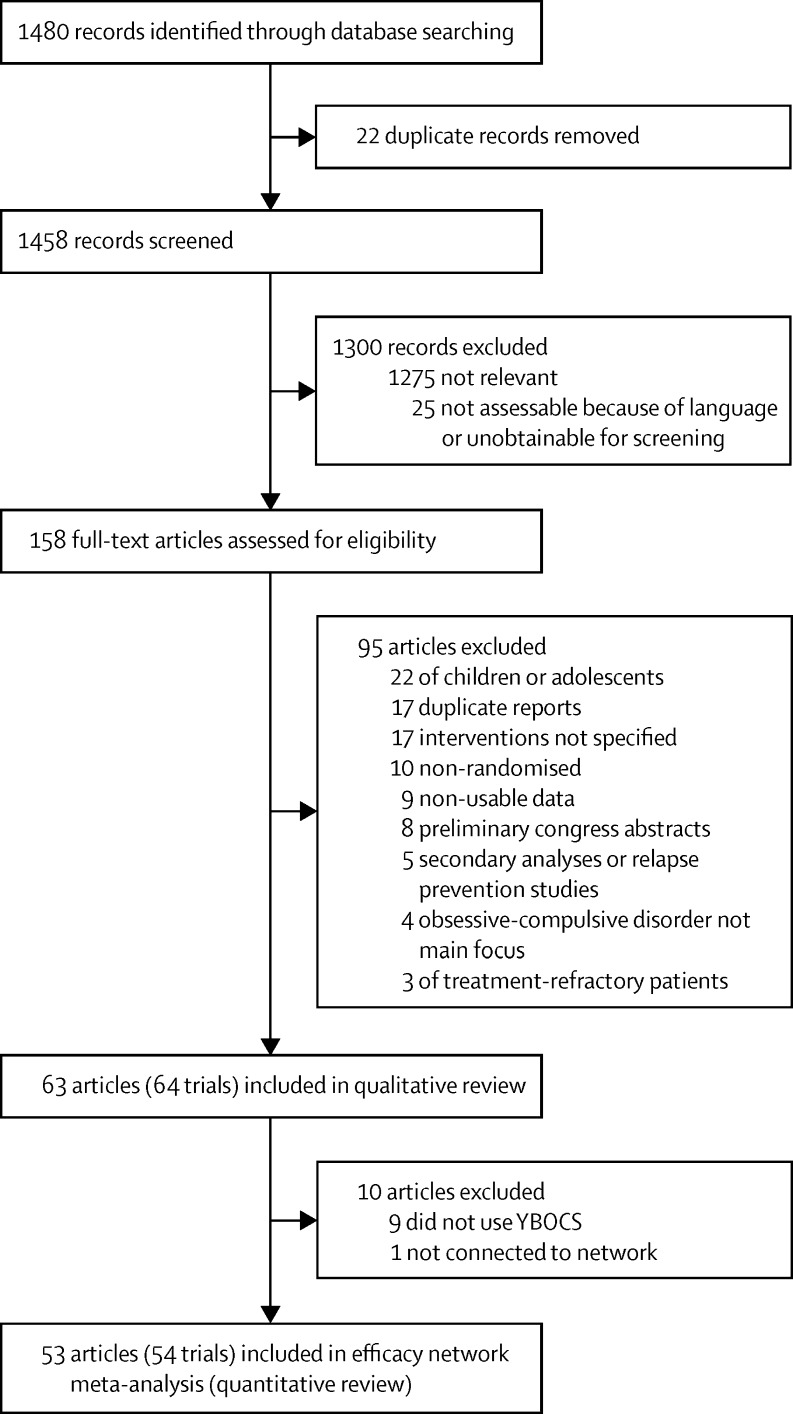
Study selection YBOCS=Yale-Brown Obsessive Compulsive Scale.

We identified 1480 articles in our search and assessed 158 (11%) full-text articles for eligibility (figure 1). We excluded 95 (60%) articles and included 64 trials reported in 63 (40%) articles^21^, ^22^, ^23^, ^24^, ^25^, ^26^, ^27^, ^28^, ^29^, ^30^, ^31^, ^32^, ^33^, ^34^, ^35^, ^36^, ^37^, ^38^, ^39^, ^40^, ^41^, ^42^, ^43^, ^44^, ^45^, ^46^, ^47^, ^48^, ^49^, ^50^, ^51^, ^52^, ^53^, ^54^, ^55^, ^56^, ^57^, ^58^, ^59^, ^60^, ^61^, ^62^, ^63^, ^64^, ^65^, ^66^, ^67^, ^68^, ^69^, ^70^, ^71^, ^72^, ^73^, ^74^, ^75^, ^76^, ^77^, ^78^, ^79^, ^80^, ^81^, ^82^, ^83^ in the qualitative review. A detailed list of the excluded studies is in the appendix. From the 63 articles eligible for inclusion in the network meta-analysis, we excluded ten (16%): nine (14%)^22^, ^32^, ^35^, ^36^, ^54^, ^62^, ^73^, ^77^, ^80^ did not use YBOCS and one (2%)^47^ was not connected to the network (details of these studies in appendix), leaving 54 trials reported in 53 (34%) articles^21^, ^23^, ^24^, ^25^, ^26^, ^27^, ^28^, ^29^, ^30^, ^31^, ^33^, ^34^, ^37^, ^38^, ^39^, ^40^, ^41^, ^42^, ^43^, ^44^, ^45^, ^46^, ^48^, ^49^, ^50^, ^51^, ^52^, ^53^, ^55^, ^56^, ^57^, ^58^, ^59^, ^60^, ^61^, ^63^, ^64^, ^65^, ^66^, ^67^, ^68^, ^69^, ^70^, ^71^, ^72^, ^74^, ^75^, ^76^, ^78^, ^79^, ^81^, ^82^, ^83^ included in the network meta-analysis (quantitative review). 7302 patients were randomly allocated in the qualitative review; however, 7014 (96%) were randomly allocated in the network meta-anlysis, with 288 (4%) excluded. Only 6652 (91%) contributed to the network meta-analysis since some trials did not report outcomes for all participants.

The 64 trials included in the qualitative review were published over a period of 33 years (1980–2012; table 1; detailed characteristics in appendix). In most psychotherapeutic trials, patients were not excluded if they were taking a stable dose of antidepressants for at least 3 months before inclusion (13 [72%] of all 18 psychotherapeutic trials and 12 [80%] of the 15 psychotherapeutic trials included in the network meta-analysis explicitly allowed antidepressants). In these trials, the proportion of patients on antidepressant medication varied, ranging from 13% to 100% and, in more than two-thirds of studies with the information available, was 45% or higher (detailed description in appendix). Patients were not allowed to make dose adjustments during trials, but no specific information was provided on how this criterion had been monitored by authors. Participants had long-standing and severe obsessive-compulsive disorder. Demographic and clinical characteristics of participants were similar across comparisons.

**Figure 2:**
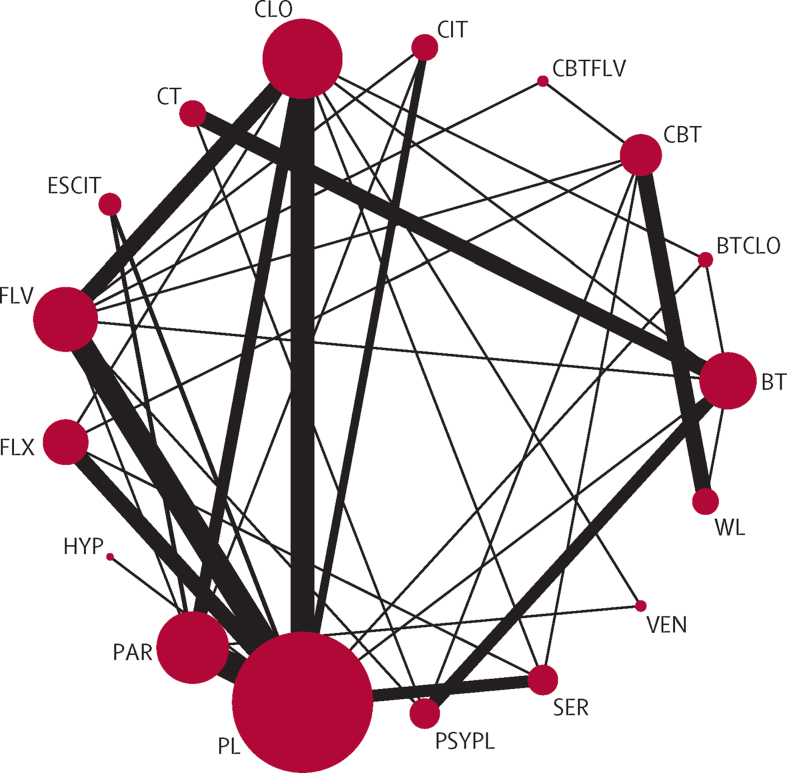
Network diagram for efficacy analysis representing direct comparisons between individual treatments The size of each circle is proportional to the number of randomly allocated participants and the width of each line is proportional to the number of trials in each direct comparison. BT=behavioural therapy. CBT=cognitive behavioural therapy. CT=cognitive therapy. BTCLO=behavioural therapy and clomipramine. CBTFLV=cognitive behavioural therapy and fluvoxamine. CIT=citalopram. CLO=clomipramine. ESCIT=escitalopram. FLV=fluvoxamine. FLX=fluoxetine. HYP=hypericum. PAR=paroxetine. PL=placebo. PSYPL=psychological placebo. SER=sertraline. VEN=venlafaxine. WL=waiting list.

The 54 trials included in the network meta-analysis (quantitative review) involved 17 different treatments grouped into 12 classes (all six SSRIs were grouped into the same class; figure 2). Overall, of the 136 unique pairwise comparisons that could be made between the 17 treatment conditions, only 37 (27%) were studied head to head in the included studies. A detailed table of the data used in the analysis is in the appendix. Six (11%) trials used a waiting list control group: five (9%) CBT studies^23^, ^31^, ^40^, ^50^, ^71^ including 157 patients, 80 (51%) of whom had been randomly allocated to CBT, and one (2%) behavioural therapy study^56^ including 40 patients, 20 (50%) of whom had been randomly allocated to behavioural therapy. The behavioural therapy trial that used the waiting list as a control group^56^ was clearly an outlier in terms of efficacy (mean YBOCS difference from waiting list at the end of study −30·87). The network meta-analysis model gave an adequate fit to the data and we identified no evidence of inconsistency (posterior mean of the residual deviance was 104·6 in the network meta-analysis assuming consistency and 105·8 assuming inconsistency compared with 107 data points). Furthermore, the deviance information criterion was similar for the models with (480·8) and without (479·1) the consistency assumption. The posterior median SD for the consistency model was 3·10 (95% credible interval 2·46–3·95), whereas for the inconsistency model, this value was reduced to 1·75 (1·18–2·53).

Most active interventions showed a significant reduction in mean YBOCS compared with drug placebo, regardless of inclusion or exclusion of trials using waiting list controls (table 2). Venlafaxine and psychological placebo both showed reductions in mean YBOCS, but they were not significant. The waiting list was the only so-called intervention that was associated with an increase in mean YBOCS compared with drug placebo. The effects of individual SSRIs were similar in magnitude. Clomipramine had a larger effect compared with placebo than did SSRIs, but the difference was not significant (table 3). All three psychotherapeutic interventions (behavioural therapy, cognitive therapy, and CBT) showed greater efficacy than did drug placebo. However, in the full analysis, CBT was less efficacious than were the other two and was not different from psychological placebo (appendix). Exclusion of studies that had used waiting list control groups led to a larger effect for CBT, which was significantly different from psychological placebo and similar to the other two psychotherapies.

In the full network, both behavioural and cognitive therapy had a larger reduction in mean YBOCS than did SSRIs as a class (table 3). CBT also had a lower mean YBOCS than SSRIs as a class, but only after excluding waiting list controlled trials. We observed similar results when comparing different types of psychotherapies with clomipramine as the reference (detailed results for all possible comparisons are shown in the appendix). However, in psychotherapeutic trials, most patients were taking stable doses of antidepressant medications for the whole duration of the trial. The same applies to the comparison between combinations of medications and psychotherapy versus psychotherapy alone as patients in these network comparisons were not in strict monotherapy (table 2, table 3). In all of these comparisons, differences were small. Excluding waiting list controlled trials, the combination of behavioural therapy with clomipramine was associated with the largest effect, but this combination has been used in only a single trial.^38^

For all 64 trials included in the qualitative review, results of the risk of bias assessment for trials with at least one drug arm (46 [72%] of 64) and those with psychotherapy arms only (18 [28%] of 64) are presented in the appendix. Sequence generation (13 [20%] of 64) and random allocation concealment (eight [13%] of 64) were specifically described (ie, low risk of bias) in few studies. In trials with psychotherapy arms, masking of participants or those delivering the intervention was not possible (seven [39%] of these 18 trials used outcome assessors who were masked to treatment allocation). In the drug only trials, specification of the double-blind method (eg, identical capsules) was described in 15 (39%) of 38 trials. Handling of incomplete outcome data with an acceptable method was reported in 28 (61%) of the 46 trials with at least one drug arm and six (33%) of the 18 trials with psychotherapy arms only. A high proportion of the trials with drug arms were sponsored by pharmaceutical companies (table 1).

For the sensitivity analyses, we used the full network (detailed results given in the appendix). In the first analysis, we included the 33 (61%) trials with low overall (<25%) and differential (<15%) attrition. This analysis led to a larger effect for CBT than in the full analysis, which was then very similar to the other two psychotherapies. In the second analysis, we included 34 (63%) trials that met the criterion of low risk of bias in the domain of incomplete outcome assessment, and the main finding was that clomipramine had a smaller effect than in the full analysis that was not different from that of SSRIs. In this analysis, we excluded all cognitive therapy trials as they had reported completers analyses. In the third analysis, we included the 17 (31%) trials that used a masked outcome assessor. Overall, results were similar to those of the full analysis, but the power was compromised because of the small sample size. We carried out separate meta-regressions to test the effect of length of trial, publication date, industry sponsorship, and inclusion of patients with current comorbid depression. The effects of these variables were small, and none were significant (appendix).

## Discussion

In this network meta-analysis, we found that several pharmacological and psychotherapeutic interventions can be considered more efficacious than is drug placebo. We found that SSRIs are generally equally efficacious, with no evidence to suggest that one drug is better than the others are. Their effect compared with placebo is statistically significant, but the estimated mean difference is generally moderate. In the full analysis, clomipramine showed a trend for a larger effect than with SSRIs that was not statistically significant. This finding contrasts with previous direct analyses, which postulated that clomipramine might be more efficacious than are SSRIs.^10^ This comparison was sensitive to studies with incomplete outcome assessment: some old clomipramine trials reported completers analyses only, and exclusion of these trials led to a lower effect for clomipramine than that of not excluding them, which was indistinguishable from that of SSRIs.

An unexpected finding was that in our main analysis, CBT had a smaller effect than that of behavioural or cognitive therapy. However, after exclusion of waiting list controlled trials, all differences between psychotherapies were not significant. The waiting list was the only so-called intervention that led to an increase in mean YBOCS score compared with drug placebo, and psychological placebo was very similar to drug placebo after exclusion of waiting list controlled trials. Research has also shown that trials using control groups with no or minimal contact with therapists usually lead to grossly overestimated effect sizes for active psychotherapeutic interventions.^18^, ^84^, ^85^ We obtained similar findings in the sensitivity analysis after exclusion of trials with high overall attrition to those from the main analysis after exclusion of waiting list controlled trials—ie, no difference between psychotherapies. The evidence for cognitive therapy mostly comes from trials that had compared it with behavioural therapy, with most of them not reporting intention-to-treat analyses, and these trials might have overestimated the effect of cognitive (and behavioural) therapy. The behavioural therapy trial that used the waiting list as a control group^56^ was clearly an outlier in terms of efficacy, and excluding it from the analysis reduced the effects for both behavioural and cognitive therapy, but not significantly. CBT has more links with other interventions and a more extensive network of trials than do cognitive and behavioural therapy and has been compared directly with several drugs in the same trial.^26^, ^71^, ^74^, ^75^ Taking all of this evidence into account, our analysis does not support the view that the three types of psychotherapy have different effects in obsessive-compulsive disorder.

Our analysis shows that all psychotherapies, either in the full dataset (for behavioural and cognitive therapy) or after exclusion of the waiting list controlled trials (for CBT), were more likely to lead to a larger effect than were medications. Some previous meta-analyses have reported similar results in favour of psychotherapy. For example, Cuijpers and colleagues^86^ examined the differential effect of pharmacotherapy and psychotherapy in major depression, dysthymia, panic disorder, social anxiety disorder, and obsessive-compulsive disorder, and reported a positive effect for psychotherapy compared with medications only for obsessive-compulsive disorder. One important limitation exists that, to our knowledge, has not been recognised before: most patients included in trials that used exclusively psychotherapeutic interventions were allowed to continue taking their antidepressant medications. Combination trials that had both psychotherapeutic and drug arms, or arms with both psychotherapy and drugs, explicitly excluded patients on antidepressant medications by design (and half of these trials were of CBT and half were of behavioural therapy). Therefore, psychotherapy trials have essentially compared different psychotherapeutic interventions in patients taking stable doses of antidepressant medications. Some evidence exists from other trials that focused exclusively on treatment-refractory patients that addition of CBT for patients with SSRI-refractory obsessive-compulsive disorder is more efficacious than is either psychological placebo^87^ or risperidone.^88^ In our analysis, although patients were symptomatic at study recruitment, what the effect would be if patients had been tapered off their antidepressant medication before randomisation is unknown because such studies have not been done. This issue has also been reported in meta-analyses of bipolar depression in which randomly allocated patients are allowed to continue using their mood stabilisers or anxiolytic medications.^89^ In any case, generalisation of these results for psychotherapeutic interventions in patients not taking concurrent antidepressant medications is difficult. Therefore, the question of what is better as monotherapy in obsessive-compulsive disorder—medications or psychotherapy—cannot be answered given the current evidence.

Our analysis has several limitations. Most studies were of short-term duration. As most of the studies that tested the efficacy of psychotherapeutic interventions included patients who were taking stable doses of antidepressant medications, generalisation of these results to patients not on medications is not possible. We were unable to test different doses of the same drug to investigate potential dose-response associations.^90^ Because of the scarce data, we could not treat alternative dosing schemes in pharmacological trials as different nodes in the network. Several old studies only reported completers analyses, including all cognitive therapy studies, limiting the usefulness of the sensitivity analysis in this domain. We did not consider the relative efficacy of the various interventions in different symptom dimensions of obsessive-compulsive disorder, and generalisation of the results in subgroups of patients with specific symptoms, such as hoarding, should be made with caution.

The results of our analysis generally support current National Institute for Health and Care Excellence guidelines.^7^ For pharmacological management, the recommendation to use SSRIs rather than clomipramine as the first-line agents is supported by our findings since SSRIs have better tolerability than does clomipramine and we identified no convincing evidence for clomipramine being more efficacious than are SSRIs. For non-pharmacological management, all three types of psychotherapy are probably more efficacious than is non-specific therapy, but evidence is limited to patients taking stable doses of antidepressant medication before initiating psychotherapy. The combined initiation of both medication and psychotherapy (either behavioural therapy or CBT) seemed an efficacious treatment. In our analysis excluding waiting list controlled trials, this combined treatment was best, but with considerable uncertainty. Given that most psychotherapeutic trials can also be considered variants of combination trials (since most patients were taking stable doses of antidepressant medications), the combination of SSRIs or clomipramine with psychotherapy is likely to offer more benefit to patients with severe illness than is monotherapy, but more research is needed than at present to support this hypothesis, including cost-effectiveness analyses.

Further research should try to differentiate more clearly than at present the effect of medications versus psychotherapy and monotherapy versus combined therapy. Trials that investigate the effect of psychotherapy should monitor use of antidepressants in included patients or recruit patients who are willing to taper off their antidepressant medication before entering randomisation. As obsessive-compulsive disorder is a very heterogeneous condition, more pragmatic trials of longer duration than have been done so far are needed to test the efficacy of existing interventions in patients encountered in daily clinical practice (including those with other comorbid conditions) and the augmenting effect of medications in addition to psychotherapy or vice versa in patients with treatment-refractory obsessive-compulsive disorder.

## Figures and Tables

**Table 1:** General characteristics of eligible studies

		**All trials (n=64)**	**Trials eligible for network meta-analysis (n=54)**
Eligible patients		7302	7014
Sample size		66 (31–159)	81 (40–168)
Eligible arms		148	127
Number of arms			
	Two	51 (80%)	42 (78%)
	Three	6 (9%)	5 (9%)
	Four	7 (11%)	7 (13%)
Year of publication			
	1980–90	10 (16%)	4 (7%)
	1991–2000	27 (42%)	23 (43%)
	2001–12	27 (42%)	27 (50%)
Type of intervention			
	Medication only	38 (59%)	33 (61%)
	Psychotherapy only	18 (28%)	15 (28%)
	Both	8 (13%)	6 (11%)
Duration (weeks)		12 (10–12)	12 (10–12)
Continent			
	North America	30 (47%)	26 (48%)
	Europe	19 (30%)	14 (26%)
	Asia	6 (9%)	6 (11%)
	Australia	3 (5%)	2 (4%)
	South America	3 (5%)	3 (6%)
	Multiple	3 (5%)	3 (6%)
Characteristics of included patients			
	Age (years)	36 (33–37)	36 (33–37)
	Disease severity (YBOCS score)	NA	25 (24–26)
	Comorbid depression	27 (42%)	19 (35%)
Pharmaceutical industry sponsorship*			
	Yes	28/46 (61%)	25/39 (64%)
	No	15/46 (33%)	12/39 (31%)
	Unclear	3/46 (7%)	2/39 (5%)
Allowed patients on antidepressant medication†			
	Yes	13/18 (72%)	12/15 (80%)
	No	4/18 (22%)	2/15 (13%)
	Unclear	1/18 (6%)	1/15 (7%)

Data are n, median (IQR), n (%), or n/N (%). YBOCS=Yale-Brown Obsessive Compulsive Scale. NA=Not applicable.

* For pharmacological trials.

† For psychotherapeutic trials.

**Table 2:** Treatment efficacy compared with drug placebo

		Number of trials (n=54)*	Number of patients (n=6652)*	**Mean YBOCS difference**	
				Full network (n=54)	Excluding waiting list controlled trials (n=48)
Drug placebo		23	1515	Reference	Reference
Waiting list		6	97	5·62 (0·91 to 10·26)	NA
Psychological placebo†		6	196	−4·15 (−8·65 to 0·49)	−1·90 (−5·62 to 1·91)
SSRIs (class effect)		37	3158	−3·49 (−5·12 to −1·81)	−3·62 (−4·89 to −2·34)
	Fluoxetine	6	633	−3·46 (−5·27 to −1·58)	−3·67 (−5·13 to −2·26)
	Fluvoxamine	13	521	−3·60 (−5·29 to −1·95)	−3·66 (−4·96 to −2·37)
	Paroxetine	8	902	−3·42 (−5·10 to −1·61)	−3·51 (−4·81 to −2·14)
	Sertraline	7	565	−3·50 (−5·30 to −1·63)	−3·68 (−5·14 to −2·30)
	Citalopram	2	311	−3·49 (−5·62 to −1·31)	−3·60 (−5·25 to −1·91)
	Escitalopram	1	226	−3·48 (−5·61 to −1·23)	−3·59 (−5·25 to −1·86)
Venlafaxine		2	98	−3·22 (−8·26 to 1·88)	−3·21 (−7·01 to 0·69)
Clomipramine		13	831	−4·72 (−6·85 to −2·60)	−4·66 (−6·26 to −3·05)
BT†		11	287	−14·48 (−18·61 to −10·23)	−10·41 (−14·04 to −6·77)
CBT†		9	231	−5·37 (−9·10 to −1·63)	−7·98 (−11·02 to −4·93)
Cognitive therapy†		6	172	−13·36 (−18·40 to −8·21)	−9·45 (−13·76 to −5·19)
Hypericum		1	30	−0·15 (−7·46 to 7·12)	−0·13 (−5·93 to 5·68)
CBT and fluvoxamine		1	6	−7·50 (−13·89 to −1·17)	−8·81 (−13·75 to −3·88)
BT and clomipramine		1	31	−12·97 (−19·18 to −6·74)	−11·68 (−16·73 to −6·65)

Data in parentheses are 95% credible intervals. YBOCS=Yale-Brown Obsessive Compulsive Scale. BT=behavioural therapy. CBT=cognitive behavioural therapy. NA=not applicable.

* Individual trials could be included in more than one treatment category.

† Several patients randomly allocated into these psychotherapeutic interventions were allowed to take stable doses of antidepressants and remain on the same dose without further adjustments.

**Table 3:** Efficacy of psychological and pharmacological interventions compared with SSRIs

	**Mean YBOCS difference in full network (n=54)**	**Mean YBOCS difference excluding waiting list controlled trials (n=48)**
SSRIs (class effect)	Reference	Reference
Clomipramine	−1·23 (−3·41 to 0·94)	−1·05 (−2·73 to 0·63)
BT*	−10·99 (−15·14 to −6·75)	−6·79 (−10·44 to −3·11)
CBT*	−1·88 (−5·52 to 1·76)	−4·36 (−7·34 to −1·40)
Cognitive therapy*	−9·87 (−14·91 to −4·74)	−5·83 (−10·17 to −1·51)
CBT and fluvoxamine	−4·03 (−10·36 to 2·21)	−5·19 (−10·09 to −0·33)
BT and clomipramine	−9·48 (−15·78 to −3·14)	−8·01 (−13·18 to −2·95)

Data in parentheses are 95% credible intervals. YBOCS=Yale-Brown Obsessive Compulsive Scale. BT=behavioural therapy. CBT=cognitive behavioural therapy.

* Several patients randomly allocated into these psychotherapeutic interventions were allowed to take stable doses of antidepressants and remain on the same dose without further adjustments.
