# Supplementary material for: Pharmacological and psychotherapeutic interventions for management of obsessive-compulsive disorder in adults: a systematic review and network meta-analysis
Source: Lancet Psychiatry. 2016 Aug;3(8):730–9. doi: 10.1016/S2215-0366(16)30069-4 (PMC4967667; doi:10.1016/S2215-0366(16)30069-4)
Supplement: Supplementary appendix [file mmc1.pdf]

THE LANCET Psychiatry

Supplementary appendix

This appendix formed part of the original submission and has been peer reviewed. We post it as supplied by the authors.

Supplement to: Skapinakis P, Caldwell DM, Hollingworth W, et al. Pharmacological and psychotherapeutic interventions for management of obsessive-compulsive disorder in adults: a systematic review and network meta-analysis. *Lancet Psychiatry* 2016; published online June 15. http://dx.doi.org/10.1016/S2215-0366(16)30069-4.

Supplementary Appendix

This appendix has been supplied by the authors to give reviewers / readers additional information about the work under consideration.

Supplement to: Skapinakis et al. Pharmacological and psychotherapeutic interventions for the management of obsessive-compulsive disorder in adults: Systematic review and Network meta-analysis

Contents

Part A: Detailed Methods

A1. OpenBugs Code used in the network-meta-analysis

Part B: Detailed Results

B1: Detailed Results of the Main Analysis with all possible comparisons between classes or individual interventions

B1.1 Detailed results of the network meta-analysis

B1.2 Detailed results of the pairwise meta-analysis (inconsistency model)

B2. Detailed results of the sensitivity analyses (versus drug placebo)

B2.1 Sensitiviy analysis 1 (Low overall attrition)

B2.2 Sensitiviy analysis 2 (Incomplete outcome data)

B2.3 Sensitivity analysis 3 (Blinding)

B3. Detailed results of the secondary analysis, excluding the waiting list controlled trials

Part C: Additional Figures

Figure A1. Risk of bias assessment

Figure A2. “Rankogram” showing the probability of rank for efficacy (MD) and acceptability (dropout rates) by intervention. Higher probability for lower rank (i.e. 1) is favourable for both outcomes

Part D: Additional Tables

Table A1. Detailed characteristics of included studies

Table A2. Data used in the network meta-analysis

Table A3 List of Excluded Trials

Table A4 Results of the meta-regression analysis

Part A: Detailed Methods

1. OpenBugs Code used in the network meta-analysis

```
#Y-BOCS random effects analysis
# Code adapted from Program 5(a)
#http://www.nicedsu.org.uk/TSD2%20General%20meta%20analysis%20correct
ed%2015April2014.pdf

model{
for (i in 1:complete){ #loop through studies reporting SD
      for(z in 1:na[i]){
            sd1[i,z]<-sd[i,z]
#calculate the mean and precision of the reported SDs
            sd1[i,z]~dnorm(mu.sd[out[i]],prec.sd[out[i]])
            }     }

for (i in complete+1:ns){#loop through remaining studies (not report
SD)
      for (z in 1:na[i]){
            sd1[i,z]~dnorm(mu.sd[out[i]],prec.sd[out[i]])
#SD is equal to estimated SD only for studies that did not report
uncertainty                   sd[i,z]<- cut(sd1[i,z])
            }     }

      for (i in 1:ns){ #loop through all studies converting SDs to
SEs
            for (z in 1:na[i]){
                  se[i,z]<-sd[i,z]/sqrt(n[i,z])
                  prec[i,z]<-pow(se[i,z],-2)
            }     }

#TSD code
for(i in 1:ns){ # LOOP THROUGH STUDIES
      w[i,1] <- 0 # adjustment for multi-arm trials is zero for
control arm
      delta[i,1] <- 0 # treatment effect is zero for control arm
      mu[i] ~ dnorm(0,.0001) # vague priors for all trial baselines
      for (k in 1:na[i]) { # LOOP THROUGH ARMS
                  y[i,k] ~ dnorm(theta[i,k],prec[i,k]) # normal
likelihood
                  theta[i,k] <- mu[i] + delta[i,k] # model for linear
predictor
                  dev[i,k] <- (y[i,k]-theta[i,k])*(y[i,k]-
theta[i,k])*prec[i,k]
            }

resdev[i] <- sum(dev[i,1:na[i]]) # summed deviance contribution

for (k in 2:na[i]) { # LOOP THROUGH ARMS
            delta[i,k] ~ dnorm(md[i,k],taud[i,k]) # trial-specific
LOR distributions
            md[i,k] <- d[t[i,k]] - d[t[i,1]] + sw[i,k] # mean of
treat effect distributions (with multi-arm trial correction)
            taud[i,k] <- tau *2*(k-1)/k # precision of treat effects
distributions
            w[i,k] <- (delta[i,k] - d[t[i,k]] + d[t[i,1]]) #
adjustment for multi-arm
            sw[i,k] <- sum(w[i,1:k-1])/(k-1) # cumulative adjustment
for multi-arm
            }
```

```
      }

totresdev <- sum(resdev[1:complete]) #Total Residual Deviance
d[1]<-0 # treatment effect is zero for reference treatment
D[1]<-0

for (i in 1:n.j1){      #vague prior for trt effects only 1 treatment
per "class"
      d[j1[i]]~dnorm(0,.0001)
      D[class[j1[i]]]<-d[j1[i]]
}

for (i in 1:n.jclass){  #trt effects when multiple treatments form a
'class'
      d[jclass[i]]~dnorm(D[class[jclass[i]]],Prec3[class[jclass[i]]])
}


D[3]~dnorm(0, 0.0001)   #vague prior for 'class' effect

Prec3[3]<- 1/(SD3*SD3)
SD3~dunif(0,10)


      # vague priors for treatment effects
      sdev ~ dunif(0,10) # vague prior for between-trial SD.
      tau <- pow(sdev,-2) # between-trial precision = (1/between-
trial variance)

for (i  in 1:2){
      mu.sd[i]~dnorm(0,.0001)I(0,)
      }
      for (i  in 1:2){
            prec.sd[i]~dgamma(.01,.01)
            }

# Ranking and probabilities for treatment and class level effects
for(k in 1:nt){
      rk[k]<-rank(d[],k)
      best[k]<-equals(rk[k],1)
            for (h in 1:nt){  prob[h,k]<-equals(rk[k],h)}  }

for (q in 1:nclass){
      rk.class[q]<-rank(D[],q)
      best.class[q]<-equals(rk.class[q],1)
            for (x in 1:nclass){
                  prob.class[x,q]<-equals(rk.class[x],q)
}     }

      # all MDs for each treatment level comparison
for (c in 1:(nt-1)) {
      for (k in (c+1):nt) {
            treat.mean.diff[c,k] <- (d[k]-d[c])  } }

# all MDs for each class level comparison
for (f in 1:(nclass-1)) {
      for (q in (f+1):nclass) {
            class.mean.diff[f,q] <- (D[q]-D[f])  } }

}
```

Part B: Detailed Results

B1 Detailed Results of the Main Analysis with all possible comparisons between classes or individual interventions

B1.1 Detailed Results of the main analysis: Network Meta-analysis

KEY:

Intervention (Class)
1 Placebo (1)
2 Waitlist (2)
3 Fluoxetine (3)
4 Fluvoxamine (3)
5 Paroxetine (3)
6 Sertraline (3)
7 Citalopram (3)
8 Venlafaxine (4)
9 Clomipramine (5)
10 Behavioural Therapy (6)
11 CBT (7)
12 Cognitive Therapy (8)
13 Hypericum (9)
14 Fluvoxamine+CBT (10)
15 BehaviouralTherapy + Clomipramine (11)
16 Escitalopram (3)
17 Psychological Placebo (12)

Network Meta-analysis: Class Effects (see key for class interventions)

	mean	sd	MC_error	val2.5pc	median	val97.5pc	start	sample
class.mean.diff[1,2]	5.62	2.378	0.02451	0.9106	5.635	10.26	50001	100000
class.mean.diff[1,3]	-3.493	0.8465	0.0126	-5.116	-3.503	-1.814	50001	100000
class.mean.diff[1,4]	-3.217	2.577	0.01525	-8.262	-3.225	1.885	50001	100000
class.mean.diff[1,5]	-4.724	1.078	0.009793	-6.851	-4.728	-2.601	50001	100000
class.mean.diff[1,6]	-14.48	2.131	0.02531	-18.61	-14.51	-10.23	50001	100000
class.mean.diff[1,7]	-5.374	1.898	0.02087	-9.098	-5.377	-1.632	50001	100000
class.mean.diff[1,8]	-13.36	2.59	0.02797	-18.4	-13.39	-8.21	50001	100000
class.mean.diff[1,9]	-0.1555	3.716	0.01768	-7.456	-0.1629	7.124	50001	100000
class.mean.diff[1,10]	-7.521	3.222	0.02186	-13.89	-7.517	-1.173	50001	100000
class.mean.diff[1,11]	-12.97	3.165	0.01717	-19.18	-12.97	-6.738	50001	100000
class.mean.diff[1,12]	-4.147	2.324	0.02623	-8.649	-4.171	0.4895	50001	100000
class.mean.diff[2,3]	-9.114	2.349	0.02079	-13.67	-9.133	-4.459	50001	100000
class.mean.diff[2,4]	-8.838	3.395	0.02372	-15.47	-8.856	-2.141	50001	100000
class.mean.diff[2,5]	-10.34	2.462	0.02028	-15.14	-10.36	-5.475	50001	100000
class.mean.diff[2,6]	-20.1	2.272	0.01427	-24.52	-20.13	-15.55	50001	100000
class.mean.diff[2,7]	-10.99	1.715	0.008719	-14.31	-11.01	-7.601	50001	100000
class.mean.diff[2,8]	-18.98	2.694	0.01744	-24.21	-19	-13.62	50001	100000
class.mean.diff[2,9]	-5.776	4.413	0.0298	-14.44	-5.784	2.911	50001	100000
class.mean.diff[2,10]	-13.14	3.501	0.01671	-19.98	-13.15	-6.262	50001	100000
class.mean.diff[2,11]	-18.59	3.719	0.02054	-25.88	-18.6	-11.23	50001	100000
class.mean.diff[2,12]	-9.768	2.455	0.01473	-14.51	-9.796	-4.857	50001	100000
class.mean.diff[3,4]	0.2759	2.553	0.01126	-4.731	0.2721	5.325	50001	100000
class.mean.diff[3,5]	-1.231	1.109	0.00638	-3.408	-1.231	0.9418	50001	100000
class.mean.diff[3,6]	-10.99	2.129	0.02277	-15.14	-11.01	-6.752	50001	100000
class.mean.diff[3,7]	-1.88	1.85	0.01643	-5.517	-1.878	1.763	50001	100000
class.mean.diff[3,8]	-9.866	2.587	0.02559	-14.91	-9.878	-4.739	50001	100000

class.mean.diff[3,9]	3.338	3.815	0.02117	-4.134	3.339	10.82	50001	100000
class.mean.diff[3,10]	-4.028	3.19	0.01734	-10.36	-4.016	2.212	50001	100000
class.mean.diff[3,11]	-9.476	3.211	0.01784	-15.78	-9.473	-3.137	50001	100000
class.mean.diff[3,12]	-0.6541	2.312	0.02379	-5.139	-0.6794	3.949	50001	100000
class.mean.diff[4,5]	-1.507	2.519	0.01091	-6.501	-1.502	3.436	50001	100000
class.mean.diff[4,6]	-11.26	3.229	0.02526	-17.57	-11.29	-4.862	50001	100000
class.mean.diff[4,7]	-2.156	3.074	0.0198	-8.191	-2.164	3.879	50001	100000
class.mean.diff[4,8]	-10.14	3.547	0.02778	-17.08	-10.16	-3.114	50001	100000
class.mean.diff[4,9]	3.062	4.517	0.02292	-5.836	3.084	11.9	50001	100000
class.mean.diff[4,10]	-4.304	4.033	0.02118	-12.26	-4.292	3.584	50001	100000
class.mean.diff[4,11]	-9.752	3.988	0.01997	-17.62	-9.746	-1.905	50001	100000
class.mean.diff[4,12]	-0.93	3.354	0.02612	-7.449	-0.9471	5.711	50001	100000
class.mean.diff[5,6]	-9.756	2.191	0.02149	-14.02	-9.771	-5.404	50001	100000
class.mean.diff[5,7]	-0.6494	2.008	0.016	-4.604	-0.642	3.293	50001	100000
class.mean.diff[5,8]	-8.635	2.638	0.02443	-13.79	-8.651	-3.385	50001	100000
class.mean.diff[5,9]	4.569	3.876	0.02024	-3.072	4.582	12.15	50001	100000
class.mean.diff[5,10]	-2.797	3.291	0.01753	-9.323	-2.794	3.677	50001	100000
class.mean.diff[5,11]	-8.245	3.177	0.01566	-14.48	-8.238	-1.984	50001	100000
class.mean.diff[5,12]	0.577	2.384	0.02248	-4.048	0.559	5.333	50001	100000
class.mean.diff[6,7]	9.106	2.089	0.01435	4.97	9.109	13.18	50001	100000
class.mean.diff[6,8]	1.12	1.561	0.008178	-1.955	1.122	4.192	50001	100000
class.mean.diff[6,9]	14.32	4.282	0.03056	5.879	14.34	22.7	50001	100000
class.mean.diff[6,10]	6.959	3.54	0.0212	-0.04063	6.976	13.87	50001	100000
class.mean.diff[6,11]	1.511	3.379	0.01985	-5.158	1.517	8.135	50001	100000
class.mean.diff[6,12]	10.33	1.547	0.006452	7.289	10.33	13.38	50001	100000
class.mean.diff[7,8]	-7.986	2.532	0.01758	-12.97	-7.981	-3.008	50001	100000
class.mean.diff[7,9]	5.218	4.167	0.02699	-2.976	5.23	13.37	50001	100000
class.mean.diff[7,10]	-2.147	3.121	0.01332	-8.307	-2.136	3.987	50001	100000
class.mean.diff[7,11]	-7.596	3.495	0.01823	-14.46	-7.606	-0.7485	50001	100000
class.mean.diff[7,12]	1.226	2.173	0.01469	-3.029	1.22	5.541	50001	100000

class.mean.diff[8,9]	13.2	4.535	0.03276	4.261	13.19	22.11	50001	100000
class.mean.diff[8,10]	5.838	3.814	0.02375	-1.666	5.839	13.33	50001	100000
class.mean.diff[8,11]	0.3902	3.698	0.02294	-6.854	0.3933	7.678	50001	100000
class.mean.diff[8,12]	9.212	1.973	0.009613	5.341	9.213	13.1	50001	100000
class.mean.diff[9,10]	-7.366	4.933	0.02833	-17.08	-7.357	2.351	50001	100000
class.mean.diff[9,11]	-12.81	4.871	0.02431	-22.35	-12.81	-3.281	50001	100000
class.mean.diff[9,12]	-3.992	4.379	0.03134	-12.55	-3.996	4.653	50001	100000
class.mean.diff[10,11]	-5.448	4.415	0.02175	-14.08	-5.445	3.286	50001	100000
class.mean.diff[10,12]	3.374	3.619	0.02194	-3.661	3.354	10.53	50001	100000
class.mean.diff[11,12]	8.822	3.563	0.02118	1.849	8.797	15.84	50001	100000

Note: Reference class appears first, for example class.mean.diff[1,3] shows the mean reduction in YBOCS of SSRIs vs placebo

Network Meta-analysis: Individual Effects

	mean	sd	MC_error	val2.5pc	median	val97.5pc	start	sample
treat.mean.diff[1,2]	5.62	2.378	0.02451	0.9106	5.635	10.26	50001	100000
treat.mean.diff[1,3]	-3.463	0.9264	0.01216	-5.268	-3.478	-1.584	50001	100000
treat.mean.diff[1,4]	-3.604	0.8429	0.01194	-5.292	-3.594	-1.947	50001	100000
treat.mean.diff[1,5]	-3.416	0.878	0.01195	-5.105	-3.431	-1.611	50001	100000
treat.mean.diff[1,6]	-3.498	0.9257	0.0124	-5.304	-3.506	-1.628	50001	100000
treat.mean.diff[1,7]	-3.488	1.065	0.01272	-5.622	-3.498	-1.309	50001	100000
treat.mean.diff[1,8]	-3.217	2.577	0.01525	-8.262	-3.225	1.885	50001	100000
treat.mean.diff[1,9]	-4.724	1.078	0.009793	-6.851	-4.728	-2.601	50001	100000
treat.mean.diff[1,10]	-14.48	2.131	0.02531	-18.61	-14.51	-10.23	50001	100000
treat.mean.diff[1,11]	-5.374	1.898	0.02087	-9.098	-5.377	-1.632	50001	100000
treat.mean.diff[1,12]	-13.36	2.59	0.02797	-18.4	-13.39	-8.21	50001	100000
treat.mean.diff[1,13]	-0.1555	3.716	0.01768	-7.456	-0.1629	7.124	50001	100000
treat.mean.diff[1,14]	-7.521	3.222	0.02186	-13.89	-7.517	-1.173	50001	100000
treat.mean.diff[1,15]	-12.97	3.165	0.01717	-19.18	-12.97	-6.738	50001	100000

treat.mean.diff[1,16]	-3.483	1.088	0.01272	-5.611	-3.498	-1.234	50001	100000
treat.mean.diff[1,17]	-4.147	2.324	0.02623	-8.649	-4.171	0.4895	50001	100000
treat.mean.diff[2,3]	-9.083	2.36	0.02061	-13.68	-9.102	-4.405	50001	100000
treat.mean.diff[2,4]	-9.225	2.329	0.02016	-13.77	-9.237	-4.628	50001	100000
treat.mean.diff[2,5]	-9.036	2.384	0.02101	-13.68	-9.06	-4.306	50001	100000
treat.mean.diff[2,6]	-9.118	2.358	0.02042	-13.73	-9.134	-4.45	50001	100000
treat.mean.diff[2,7]	-9.108	2.451	0.0212	-13.89	-9.127	-4.253	50001	100000
treat.mean.diff[2,8]	-8.838	3.395	0.02372	-15.47	-8.856	-2.141	50001	100000
treat.mean.diff[2,9]	-10.34	2.462	0.02028	-15.14	-10.36	-5.475	50001	100000
treat.mean.diff[2,10]	-20.1	2.272	0.01427	-24.52	-20.13	-15.55	50001	100000
treat.mean.diff[2,11]	-10.99	1.715	0.008719	-14.31	-11.01	-7.601	50001	100000
treat.mean.diff[2,12]	-18.98	2.694	0.01744	-24.21	-19	-13.62	50001	100000
treat.mean.diff[2,13]	-5.776	4.413	0.0298	-14.44	-5.784	2.911	50001	100000
treat.mean.diff[2,14]	-13.14	3.501	0.01671	-19.98	-13.15	-6.262	50001	100000
treat.mean.diff[2,15]	-18.59	3.719	0.02054	-25.88	-18.6	-11.23	50001	100000
treat.mean.diff[2,16]	-9.103	2.457	0.02115	-13.9	-9.118	-4.223	50001	100000
treat.mean.diff[2,17]	-9.768	2.455	0.01473	-14.51	-9.796	-4.857	50001	100000
treat.mean.diff[3,4]	-0.1412	0.8752	0.004168	-2.196	-0.04312	1.62	50001	100000
treat.mean.diff[3,5]	0.04729	0.8874	0.003442	-1.843	0.009691	2.033	50001	100000
treat.mean.diff[3,6]	-0.03457	0.8854	0.00315	-2.019	-0.00726	1.857	50001	100000
treat.mean.diff[3,7]	-0.02461	1.042	0.004036	-2.315	-0.00442	2.256	50001	100000
treat.mean.diff[3,8]	0.2457	2.602	0.01119	-4.871	0.2425	5.377	50001	100000
treat.mean.diff[3,9]	-1.261	1.182	0.006338	-3.63	-1.254	1.055	50001	100000
treat.mean.diff[3,10]	-11.02	2.155	0.0226	-15.23	-11.04	-6.742	50001	100000
treat.mean.diff[3,11]	-1.911	1.853	0.01622	-5.549	-1.902	1.728	50001	100000
treat.mean.diff[3,12]	-9.896	2.606	0.02546	-14.98	-9.912	-4.757	50001	100000
treat.mean.diff[3,13]	3.308	3.832	0.02092	-4.217	3.312	10.81	50001	100000
treat.mean.diff[3,14]	-4.058	3.21	0.01732	-10.43	-4.039	2.225	50001	100000
treat.mean.diff[3,15]	-9.506	3.231	0.01754	-15.86	-9.5	-3.137	50001	100000
treat.mean.diff[3,16]	-0.01967	1.048	0.003805	-2.314	-0.0041	2.276	50001	100000

treat.mean.diff[3,17]	-0.6843	2.33	0.02355	-5.218	-0.7018	3.94	50001	100000
treat.mean.diff[4,5]	0.1885	0.8269	0.004488	-1.409	0.06661	2.146	50001	100000
treat.mean.diff[4,6]	0.1066	0.8689	0.003919	-1.676	0.02994	2.116	50001	100000
treat.mean.diff[4,7]	0.1166	0.9929	0.004682	-1.94	0.03256	2.417	50001	100000
treat.mean.diff[4,8]	0.3869	2.571	0.0118	-4.653	0.377	5.462	50001	100000
treat.mean.diff[4,9]	-1.12	1.083	0.005907	-3.24	-1.131	1.036	50001	100000
treat.mean.diff[4,10]	-10.88	2.099	0.02222	-14.96	-10.91	-6.69	50001	100000
treat.mean.diff[4,11]	-1.769	1.831	0.01591	-5.365	-1.776	1.866	50001	100000
treat.mean.diff[4,12]	-9.755	2.557	0.0251	-14.73	-9.778	-4.697	50001	100000
treat.mean.diff[4,13]	3.449	3.816	0.02111	-4.032	3.454	10.95	50001	100000
treat.mean.diff[4,14]	-3.917	3.147	0.01687	-10.14	-3.912	2.281	50001	100000
treat.mean.diff[4,15]	-9.365	3.198	0.01756	-15.62	-9.371	-3.02	50001	100000
treat.mean.diff[4,16]	0.1215	1.012	0.004475	-1.937	0.02962	2.493	50001	100000
treat.mean.diff[4,17]	-0.5431	2.283	0.02332	-4.964	-0.566	4.005	50001	100000
treat.mean.diff[5,6]	-0.08186	0.8859	0.003587	-2.096	-0.0227	1.769	50001	100000
treat.mean.diff[5,7]	-0.0719	0.9897	0.004086	-2.309	-0.01626	2.029	50001	100000
treat.mean.diff[5,8]	0.1984	2.501	0.01046	-4.704	0.1935	5.125	50001	100000
treat.mean.diff[5,9]	-1.309	1.101	0.005826	-3.508	-1.304	0.848	50001	100000
treat.mean.diff[5,10]	-11.06	2.156	0.023	-15.27	-11.08	-6.784	50001	100000
treat.mean.diff[5,11]	-1.958	1.898	0.0166	-5.704	-1.955	1.762	50001	100000
treat.mean.diff[5,12]	-9.944	2.61	0.02578	-15.06	-9.961	-4.784	50001	100000
treat.mean.diff[5,13]	3.26	3.821	0.02081	-4.243	3.263	10.76	50001	100000
treat.mean.diff[5,14]	-4.105	3.218	0.0176	-10.49	-4.092	2.212	50001	100000
treat.mean.diff[5,15]	-9.554	3.214	0.01765	-15.88	-9.547	-3.232	50001	100000
treat.mean.diff[5,16]	-0.06696	0.9761	0.003684	-2.261	-0.01811	2.024	50001	100000
treat.mean.diff[5,17]	-0.7316	2.342	0.02399	-5.301	-0.7494	3.927	50001	100000
treat.mean.diff[6,7]	0.009959	1.039	0.003995	-2.271	0.001729	2.285	50001	100000
treat.mean.diff[6,8]	0.2803	2.596	0.01146	-4.789	0.2729	5.417	50001	100000
treat.mean.diff[6,9]	-1.227	1.173	0.006392	-3.552	-1.224	1.095	50001	100000
treat.mean.diff[6,10]	-10.98	2.153	0.02246	-15.16	-11.01	-6.708	50001	100000

treat.mean.diff[6,11]	-1.876	1.859	0.01605	-5.532	-1.881	1.798	50001	100000
treat.mean.diff[6,12]	-9.862	2.604	0.0253	-14.93	-9.871	-4.706	50001	100000
treat.mean.diff[6,13]	3.342	3.835	0.02118	-4.181	3.344	10.86	50001	100000
treat.mean.diff[6,14]	-4.023	3.209	0.01722	-10.39	-4.009	2.274	50001	100000
treat.mean.diff[6,15]	-9.472	3.23	0.01747	-15.82	-9.468	-3.113	50001	100000
treat.mean.diff[6,16]	0.0149	1.044	0.003616	-2.26	0.001961	2.338	50001	100000
treat.mean.diff[6,17]	-0.6497	2.333	0.02343	-5.19	-0.6678	3.987	50001	100000
treat.mean.diff[7,8]	0.2703	2.644	0.01148	-4.932	0.262	5.48	50001	100000
treat.mean.diff[7,9]	-1.237	1.304	0.007012	-3.847	-1.229	1.338	50001	100000
treat.mean.diff[7,10]	-10.99	2.24	0.0231	-15.36	-11.01	-6.56	50001	100000
treat.mean.diff[7,11]	-1.886	1.982	0.01693	-5.806	-1.884	2.029	50001	100000
treat.mean.diff[7,12]	-9.872	2.677	0.02598	-15.12	-9.893	-4.573	50001	100000
treat.mean.diff[7,13]	3.332	3.87	0.02124	-4.254	3.332	10.93	50001	100000
treat.mean.diff[7,14]	-4.033	3.265	0.01785	-10.52	-4.013	2.361	50001	100000
treat.mean.diff[7,15]	-9.482	3.281	0.01792	-15.91	-9.484	-3.001	50001	100000
treat.mean.diff[7,16]	0.004941	1.13	0.003898	-2.469	-3.77E-04	2.509	50001	100000
treat.mean.diff[7,17]	-0.6597	2.418	0.02409	-5.361	-0.6761	4.163	50001	100000
treat.mean.diff[8,9]	-1.507	2.519	0.01091	-6.501	-1.502	3.436	50001	100000
treat.mean.diff[8,10]	-11.26	3.229	0.02526	-17.57	-11.29	-4.862	50001	100000
treat.mean.diff[8,11]	-2.156	3.074	0.0198	-8.191	-2.164	3.879	50001	100000
treat.mean.diff[8,12]	-10.14	3.547	0.02778	-17.08	-10.16	-3.114	50001	100000
treat.mean.diff[8,13]	3.062	4.517	0.02292	-5.836	3.084	11.9	50001	100000
treat.mean.diff[8,14]	-4.304	4.033	0.02118	-12.26	-4.292	3.584	50001	100000
treat.mean.diff[8,15]	-9.752	3.988	0.01997	-17.62	-9.746	-1.905	50001	100000
treat.mean.diff[8,16]	-0.2654	2.646	0.0116	-5.501	-0.2571	4.944	50001	100000
treat.mean.diff[8,17]	-0.93	3.354	0.02612	-7.449	-0.9471	5.711	50001	100000
treat.mean.diff[9,10]	-9.756	2.191	0.02149	-14.02	-9.771	-5.404	50001	100000
treat.mean.diff[9,11]	-0.6494	2.008	0.016	-4.604	-0.642	3.293	50001	100000
treat.mean.diff[9,12]	-8.635	2.638	0.02443	-13.79	-8.651	-3.385	50001	100000
treat.mean.diff[9,13]	4.569	3.876	0.02024	-3.072	4.582	12.15	50001	100000

treat.mean.diff[9,14]	-2.797	3.291	0.01753	-9.323	-2.794	3.677	50001	100000
treat.mean.diff[9,15]	-8.245	3.177	0.01566	-14.48	-8.238	-1.984	50001	100000
treat.mean.diff[9,16]	1.242	1.316	0.006932	-1.352	1.236	3.89	50001	100000
treat.mean.diff[9,17]	0.577	2.384	0.02248	-4.048	0.559	5.333	50001	100000
treat.mean.diff[10,11]	9.106	2.089	0.01435	4.97	9.109	13.18	50001	100000
treat.mean.diff[10,12]	1.12	1.561	0.008178	-1.955	1.122	4.192	50001	100000
treat.mean.diff[10,13]	14.32	4.282	0.03056	5.879	14.34	22.7	50001	100000
treat.mean.diff[10,14]	6.959	3.54	0.0212	-0.04063	6.976	13.87	50001	100000
treat.mean.diff[10,15]	1.511	3.379	0.01985	-5.158	1.517	8.135	50001	100000
treat.mean.diff[10,16]	11	2.241	0.02308	6.548	11.01	15.4	50001	100000
treat.mean.diff[10,17]	10.33	1.547	0.006452	7.289	10.33	13.38	50001	100000
treat.mean.diff[11,12]	-7.986	2.532	0.01758	-12.97	-7.981	-3.008	50001	100000
treat.mean.diff[11,13]	5.218	4.167	0.02699	-2.976	5.23	13.37	50001	100000
treat.mean.diff[11,14]	-2.147	3.121	0.01332	-8.307	-2.136	3.987	50001	100000
treat.mean.diff[11,15]	-7.596	3.495	0.01823	-14.46	-7.606	-0.7485	50001	100000
treat.mean.diff[11,16]	1.891	1.985	0.0168	-2.03	1.891	5.813	50001	100000
treat.mean.diff[11,17]	1.226	2.173	0.01469	-3.029	1.22	5.541	50001	100000
treat.mean.diff[12,13]	13.2	4.535	0.03276	4.261	13.19	22.11	50001	100000
treat.mean.diff[12,14]	5.838	3.814	0.02375	-1.666	5.839	13.33	50001	100000
treat.mean.diff[12,15]	0.3902	3.698	0.02294	-6.854	0.3933	7.678	50001	100000
treat.mean.diff[12,16]	9.877	2.678	0.02587	4.591	9.889	15.14	50001	100000
treat.mean.diff[12,17]	9.212	1.973	0.009613	5.341	9.213	13.1	50001	100000
treat.mean.diff[13,14]	-7.366	4.933	0.02833	-17.08	-7.357	2.351	50001	100000
treat.mean.diff[13,15]	-12.81	4.871	0.02431	-22.35	-12.81	-3.281	50001	100000
treat.mean.diff[13,16]	-3.327	3.874	0.02134	-10.94	-3.331	4.227	50001	100000
treat.mean.diff[13,17]	-3.992	4.379	0.03134	-12.55	-3.996	4.653	50001	100000
treat.mean.diff[14,15]	-5.448	4.415	0.02175	-14.08	-5.445	3.286	50001	100000
treat.mean.diff[14,16]	4.038	3.274	0.01775	-2.386	4.023	10.51	50001	100000
treat.mean.diff[14,17]	3.374	3.619	0.02194	-3.661	3.354	10.53	50001	100000
treat.mean.diff[15,16]	9.487	3.285	0.01803	3.003	9.481	15.93	50001	100000

treat.mean.diff[15,17]	8.822	3.563	0.02118	1.849	8.797	15.84	50001	100000
treat.mean.diff[16,17]	-0.6646	2.418	0.0241	-5.384	-0.6767	4.141	50001	100000

Note: Reference class appears first, for example treat.mean.diff[1,3] shows the mean reduction in YBOCS of fluoxetine vs placebo

B1.2 Detailed Results of the Pairwise meta-analysis (Inconsistency Model)

d	mean	sd	MC_error	val2.5pc	median	val97.5pc	start	sample
d[1,3]	-2.656	1.064	0.006615	-4.722	-2.666	-0.5367	60001	120000
d[1,4]	-3.577	0.9644	0.005258	-5.513	-3.566	-1.704	60001	120000
d[1,5]	-2.839	0.8353	0.004452	-4.48	-2.844	-1.174	60001	120000
d[1,6]	-2.85	1.188	0.006132	-5.182	-2.854	-0.503	60001	120000
d[1,7]	-3.653	1.32	0.008286	-6.255	-3.651	-1.056	60001	120000
d[1,9]	-6.278	0.9656	0.004634	-8.154	-6.291	-4.34	60001	120000
d[1,10]	-11.76	2.598	0.0167	-16.87	-11.77	-6.617	60001	120000
d[1,13]	-0.0795	2.658	0.01658	-5.297	-0.08029	5.112	60001	120000
d[1,15]	-12.25	2.599	0.01664	-17.29	-12.26	-7.094	60001	120000
d[1,16]	-3.281	1.563	0.008429	-6.38	-3.275	-0.1997	60001	120000
d[2,10]	-30.86	1.945	0.00709	-34.72	-30.86	-27	60001	120000
d[2,11]	-7.392	1.452	0.0107	-10.25	-7.395	-4.542	60001	120000
d[3,6]	0.1054	2.22	0.009702	-4.27	0.106	4.466	60001	120000
d[3,9]	-1.422	2.862	0.0203	-7.063	-1.414	4.208	60001	120000
d[3,11]	-0.3179	2.244	0.01055	-4.766	-0.3078	4.083	60001	120000
d[4,5]	5.399	4.21	0.0448	-2.866	5.41	13.59	60001	120000
d[4,7]	3.603	4.507	0.05032	-5.237	3.594	12.44	60001	120000
d[4,9]	-0.1027	1.331	0.007964	-2.735	-0.1039	2.515	60001	120000
d[4,10]	-7.277	3.785	0.05031	-14.74	-7.247	0.05766	60001	120000
d[4,11]	-9.656	2.408	0.01619	-14.4	-9.656	-4.914	60001	120000
d[4,14]	-8.149	2.426	0.0168	-12.92	-8.147	-3.367	60001	120000
d[4,17]	8.227	3.961	0.05129	0.4291	8.253	15.93	60001	120000

d[5,7]	not estimable							
d[5,8]	0.6068	2.106	0.008706	-3.561	0.6089	4.744	60001	120000
d[5,9]	1.06	2.109	0.009106	-3.096	1.06	5.213	60001	120000
d[5,16]	not estimable							
d[6,9]	2.581	2.123	0.009151	-1.611	2.584	6.762	60001	120000
d[6,11]	-3.44	2.711	0.01678	-8.768	-3.438	1.924	60001	120000
d[8,9]	-1.071	2.502	0.0164	-5.988	-1.08	3.865	60001	120000
d[9,10]	not estimable							
d[9,15]	not estimable							
d[10,12]	-0.3039	1.246	0.008738	-2.747	-0.3015	2.147	60001	120000
d[10,15]	not estimable							
d[10,17]	8.424	1.437	0.008111	5.641	8.405	11.31	60001	120000
d[11,14]	not estimable							
d[11,17]	5.937	2.105	0.01018	1.741	5.942	10.06	60001	120000
d[12,17]	2.673	2.301	0.01049	-1.86	2.686	7.185	60001	120000

Note: Reference class appears first, for example d[1,3] shows the mean reduction in YBOCS of fluoxetine vs placebo

B2 Detailed Results of the Sensitivity Analyses

B2.1 Detailed Results of the Sensitivity Analysis 1 (Low overall attrition)

Raw Data Used

Studyid	y[,1]	n[,1]	sd[,1]	y[,2]	n[,2]	sd[,2]	y[,3]	n[,3]	sd[,3]	y[,4]	n[,4]	sd[,4]	na[]	out[]	t[,1]	t[,2]	t[,3]	t[,4]
#Albert2001	18.36	25	7.11	17.3	40	6.15	NA	NA	NA	NA	NA	NA	2	2	8	9	NA	NA
#Anderson2007	23.5	14	6.4	16.7	17	6.8	NA	NA	NA	NA	NA	NA	2	2	2	11	NA	NA
#Andersson2012	12.94	49	6.26	18.88	51	4.18	NA	NA	NA	NA	NA	NA	2	2	11	17	NA	NA
#Belloch2008	8.31	13	8.75	6.8	16	3.55	NA	NA	NA	NA	NA	NA	2	2	10	12	NA	NA
#CCSG1	25.11	108	6.34	16.23	102	7.37	NA	NA	NA	NA	NA	NA	2	2	1	9	NA	NA
#CCSG2	25.59	119	5.78	14.7	120	7.45	NA	NA	NA	NA	NA	NA	2	2	1	9	NA	NA
#Cordioli2003	23.2	24	5.5	15.1	23	7.8	NA	NA	NA	NA	NA	NA	2	2	2	11	NA	NA
#Cottraux2001	-12.1	30	7.8	-12.5	30	8.2	NA	NA	NA	NA	NA	NA	2	1	10	12	NA	NA
#Denys2003	-7.8	72	5.4	-7.2	73	7.5	NA	NA	NA	NA	NA	NA	2	1	5	8	NA	NA
#Greist2002	17.6	55	6.2	24.1	66	6.7	NA	NA	NA	NA	NA	NA	2	2	10	17	NA	NA
#GSK526	-14.26	72	6.33	-13.19	69	6.48	NA	NA	NA	NA	NA	NA	2	1	5	9	NA	NA
#Jaurrieta2008	24.6	19	8.9	17.8	19	8.4	NA	NA	NA	NA	NA	NA	2	2	2	11	NA	NA
#Jenike1990a	22.3	9	7.8	20.6	10	9.2	NA	NA	NA	NA	NA	NA	2	2	1	6	NA	NA
#Jenike1990b	21.8	20	7.6	18.8	18	4	NA	NA	NA	NA	NA	NA	2	2	1	4	NA	NA
#Jenike1997	18.7	18	6.1	16.2	19	6.3	NA	NA	NA	NA	NA	NA	2	2	1	3	NA	NA
#Kamijima2004	20.3	94	7.38	15.8	94	8.09	NA	NA	NA	NA	NA	NA	2	2	1	5	NA	NA
#Lindsay1997	11	9	3.81	25.89	9	5.8	NA	NA	NA	NA	NA	NA	2	2	10	17	NA	NA
#Milanfranchi1997	18.4	13	9.2	16.5	12	11	NA	NA	NA	NA	NA	NA	2	2	4	9	NA	NA
#Montgomery2001	-5.6	101	6.9	-8.4	102	7.3	-8.9	98	7	-10.4	100	6.9	4	1	1	7	7	7
#Mundo1997	16.2	10	8.9	21.6	9	7.6	19.8	11	10.1	NA	NA	NA	3	2	4	5	7	NA

#Nakatani2005	20.2	10	9.4	12.9	10	4.9	28.4	8	5.5	NA	NA	NA	3	2	4	10	17	NA
#O'Konnor1999	17.5	6	4	13.3	6	8.6	NA	NA	NA	NA	NA	NA	2	2	2	11	NA	NA
#Shareh2010	16.66	6	3.2	7	7	2.38	8.5	6	2.42	NA	NA	NA	3	2	4	11	14	NA
#Stein2007	-8.46	113	8.08	-11.67	116	8.40	-11.43	112	8.25	-12.14	114	8.22	4	1	1	5	16	16
#Tollefson1994	-0.8	89	5.66	-5.44	266	7.88	NA	NA	NA	NA	NA	NA	2	1	1	3	NA	NA
#Lopez-Ibor1996	-7.5	30	9.29	-8.9	24	7.13	NA	NA	NA	NA	NA	NA	2	1	3	9	NA	NA
#Van Oppen1995	17.9	29	9	13.4	28	9.4	NA	NA	NA	NA	NA	NA	2	2	10	12	NA	NA
#Whittal2010	6.43	37	4.77	9.1	30	6.48	NA	NA	NA	NA	NA	NA	2	2	12	17	NA	NA
#Chouinard1990	-1.48	44	NA	-3.79	43	NA	NA	NA	NA	NA	NA	NA	2	1	1	6	NA	NA
#Fals-Stewart1993	-8.1	31	NA	-1.8	32	NA	NA	NA	NA	NA	NA	NA	2	1	10	17	NA	NA
#Hollander2003b	-3.33	89	NA	-4.14	88	NA	-6.35	86	NA	-7.34	85	NA	4	1	1	5	5	5
#Mundo2001	-12.2	115	NA	-12	112	NA	NA	NA	NA	NA	NA	NA	2	1	4	9	NA	NA
#Sousa2006	-7.36	25	NA	-10.8	25	NA	NA	NA	NA	NA	NA	NA	2	1	6	11	NA	NA

Key

Intervention (Class)
1 Placebo (1)
2 Waitlist (2)
3 Fluoxetine(3)
4 Fluvoxamine(3)
5 Paroxetine (3)
6 Sertraline (3)
7 Citalopram (3)
8 Venlafaxine (4)

9 Clomipramine(5)
10 Behavioural Therapy (6)
11 CBT (7)
12 Cognitive Therapy (8)
13 CBT + Fluvoxamine(9)
14 Escitalopram (3)
15 Psychological Placebo (10)

Class Effects vs. Placebo (see key for class interventions)

	mean	sd	MC_error	val2.5pc	median	val97.5pc	start	sample
class.mean.diff[1,2]	-3.317	2.875	0.03854	-8.983	-3.324	2.38	50001	100000
class.mean.diff[1,3]	-4.091	1.005	0.0165	-6.066	-4.092	-2.061	50001	100000
class.mean.diff[1,4]	-4.319	2.234	0.01698	-8.719	-4.321	0.1204	50001	100000
class.mean.diff[1,5]	-6.419	1.284	0.01173	-8.932	-6.435	-3.852	50001	100000
class.mean.diff[1,6]	-11.04	2.971	0.0506	-16.84	-11.04	-5.186	50001	100000
class.mean.diff[1,7]	-10.13	2.241	0.03435	-14.52	-10.15	-5.687	50001	100000
class.mean.diff[1,8]	-10.63	3.282	0.05325	-17.08	-10.62	-4.16	50001	100000
class.mean.diff[1,9]	-10.31	2.939	0.0301	-16.14	-10.31	-4.521	50001	100000
class.mean.diff[1,10]	-2.851	2.811	0.04821	-8.334	-2.868	2.771	50001	100000

Note: Reference class appears first, for example class.mean.diff[1,3] shows the mean reduction in YBOCS of SSRIs vs placebo

Individual Effects vs. Placebo (see key for individual interventions)

	mean	sd	MC_error	val2.5pc	median	val97.5pc	start	sample
treat.mean.diff[1,2]	-3.317	2.875	0.03854	-8.983	-3.324	2.38	50001	100000

treat.mean.diff[1,3]	-4.103	1.11	0.01554	-6.307	-4.106	-1.837	50001	100000
treat.mean.diff[1,4]	-4.259	1.141	0.0184	-6.642	-4.224	-1.997	50001	100000
treat.mean.diff[1,5]	-4.096	0.9845	0.01555	-6.032	-4.098	-2.095	50001	100000
treat.mean.diff[1,6]	-4.048	1.193	0.01704	-6.413	-4.065	-1.62	50001	100000
treat.mean.diff[1,7]	-4.009	1.149	0.01604	-6.247	-4.031	-1.633	50001	100000
treat.mean.diff[1,8]	-4.319	2.234	0.01698	-8.719	-4.321	0.1204	50001	100000
treat.mean.diff[1,9]	-6.419	1.284	0.01173	-8.932	-6.435	-3.852	50001	100000
treat.mean.diff[1,10]	-11.04	2.971	0.0506	-16.84	-11.04	-5.186	50001	100000
treat.mean.diff[1,11]	-10.13	2.241	0.03435	-14.52	-10.15	-5.687	50001	100000
treat.mean.diff[1,12]	-10.63	3.282	0.05325	-17.08	-10.62	-4.16	50001	100000
treat.mean.diff[1,13]	-10.31	2.939	0.0301	-16.14	-10.31	-4.521	50001	100000
treat.mean.diff[1,14]	-4.031	1.169	0.01598	-6.304	-4.05	-1.609	50001	100000
treat.mean.diff[1,15]	-2.851	2.811	0.04821	-8.334	-2.868	2.771	50001	100000

Note: Reference class appears first, for example treat.mean.diff[1,3] shows the mean reduction in YBOCS of fluoxetine vs placebo

B2.2 Detailed Results of the Sensitivity Analysis 2 (Incomplete outcome data)

Raw Data Used

Studyid	y[,1]	n[,1]	sd[,1]	y[,2]	n[,2]	sd[,2]	y[,3]	n[,3]	sd[,3]	y[,4]	n[,4]	sd[,4]	na[]	out[]	t[,1]	t[,2]	t[,3]	t[,4]
#Anderson2007	23.5	14	6.4	16.7	17	6.8	NA	NA	NA	NA	NA	NA	2	2	2	11	NA	NA
#Andersson2012	12.94	49	6.26	18.88	51	4.18	NA	NA	NA	NA	NA	NA	2	2	11	15	NA	NA
#Belotto-Silva2012	20.29	88	8.05	19.97	70	8.48	NA	NA	NA	NA	NA	NA	2	2	3	11	NA	NA
#Bergeron2002	-9.7	72	7.7	-9.6	76	7.9	NA	NA	NA	NA	NA	NA	2	1	3	6	NA	NA
#Cordioli2003	23.2	24	5.5	15.1	23	7.8	NA	NA	NA	NA	NA	NA	2	2	2	11	NA	NA
#Denys2003	-7.8	72	5.4	-7.2	73	7.5	NA	NA	NA	NA	NA	NA	2	1	5	8	NA	NA
#Foa2005	22.2	26	6.4	18.2	36	7.8	11	29	7.9	10.5	31	8.2	4	2	1	9	10	13
#Freeston1997	22	14	6	12.2	15	9.6	NA	NA	NA	NA	NA	NA	2	2	2	11	NA	NA
#Goodman1989	28	21	7	19.4	21	7	NA	NA	NA	NA	NA	NA	2	2	1	4	NA	NA
#Goodman1996	-1.71	78	4.88	-3.95	78	6.28	NA	NA	NA	NA	NA	NA	2	1	1	4	NA	NA
#Greist2002	17.6	55	6.2	24.1	66	6.7	NA	NA	NA	NA	NA	NA	2	2	10	15	NA	NA
#GSK118	-4.61	75	7.53	-5.61	79	7.47	-7.73	78	7.42	NA	NA	NA	3	1	1	5	9	NA
#Hollander2003	-5.6	120	7.67	-8.5	117	7.57	NA	NA	NA	NA	NA	NA	2	1	1	4	NA	NA
#Jaurrieta2008	24.6	19	8.9	17.8	19	8.4	NA	NA	NA	NA	NA	NA	2	2	2	11	NA	NA
#Jenike1990a	22.3	9	7.8	20.6	10	9.2	NA	NA	NA	NA	NA	NA	2	2	1	6	NA	NA
#Jenike1997	18.7	18	6.1	16.2	19	6.3	NA	NA	NA	NA	NA	NA	2	2	1	3	NA	NA
#Kamijima2004	20.3	94	7.38	15.8	94	8.09	NA	NA	NA	NA	NA	NA	2	2	1	5	NA	NA
#Kobak2005	19.87	30	7.46	19.75	30	7.46	NA	NA	NA	NA	NA	NA	2	2	1	12	NA	NA
#Koran1996	17.8	34	7.7	17	39	8.55	NA	NA	NA	NA	NA	NA	2	2	4	9	NA	NA
#Montgomery1993	-3.7	56	5.98	-5.13	52	6.41	-4.76	52	6.89	-6.07	54	6.92	4	1	1	3	3	3
#Montgomery2001	-5.6	101	6.9	-8.4	102	7.3	-8.9	98	7	-10.4	100	6.9	4	1	1	7	7	7
#Mundo1997	16.2	10	8.9	21.6	9	7.6	19.8	11	10.1	NA	NA	NA	3	2	4	5	7	NA
#Nakajima1996	-1.9	33	7.2	-7.1	60	7.03	NA	NA	NA	NA	NA	NA	2	1	1	4	NA	NA

#Stein2007	-8.46	113	8.08	-11.67	116	8.40	-11.43	112	8.25	-12.14	114	8.22	4	1	1	5	14	14
#Tollefson1994	-0.8	89	5.66	-5.44	266	7.88	NA	NA	NA	NA	NA	NA	2	1	1	3	NA	NA
#Lopez-Ibor1996	-7.5	30	9.29	-8.9	24	7.13	NA	NA	NA	NA	NA	NA	2	1	3	9	NA	NA
#Zohar1996	-4.2	99	7.2	-6.4	201	7.1	-7	99	6.8	NA	NA	NA	3	1	1	5	9	NA
#Kronig1999	-4.14	79	NA	-8.5	85	NA	NA	NA	NA	NA	NA	NA	2	1	1	6	NA	NA
#Bisserbe1997	-14.3	86	NA	-11.71	81	NA	NA	NA	NA	NA	NA	NA	2	1	6	9	NA	NA
#Chouinard1990	-1.48	44	NA	-3.79	43	NA	NA	NA	NA	NA	NA	NA	2	1	1	6	NA	NA
#Freeman1994	-8.6	28	NA	-7.8	19	NA	NA	NA	NA	NA	NA	NA	2	1	4	9	NA	NA
#Hollander2003b	-3.33	89	NA	-4.14	88	NA	-6.35	86	NA	-7.34	85	NA	4	1	1	5	5	5
#Mundo2001	-12.2	115	NA	-12	112	NA	NA	NA	NA	NA	NA	NA	2	1	4	9	NA	NA
#Sousa2006	-7.36	25	NA	-10.8	25	NA	NA	NA	NA	NA	NA	NA	2	1	6	11	NA	NA

Key

Intervention (Class)
1 Placebo (1)
2 Waitlist (2)
3 Fluoxetine(3)
4 Fluvoxamine (3)
5 Paroxetine (3)
6 Sertraline (3)
7 Citalopram (3)
8 Venlafaxine (4)
9 Clomipramine (5)
10 Behavioural Therapy [BT] (6)
11 CBT (7)

12 Hypericum (8)
13 BT + Clomipramine (9)
14 Escitalopram (3)
15 Psychological Placebo (10)

Class Effects vs. Placebo (see key for class interventions)

	mean	sd	MC_error	val2.5pc	median	val97.5pc	start	sample
class.mean.diff[1,2]	2.063	1.806	0.03318	-1.509	2.067	5.611	50001	100000
class.mean.diff[1,3]	-3.332	0.4591	0.006652	-4.25	-3.329	-2.456	50001	100000
class.mean.diff[1,4]	-2.458	1.533	0.01317	-5.492	-2.461	0.5665	50001	100000
class.mean.diff[1,5]	-3.156	0.6228	0.008235	-4.392	-3.151	-1.95	50001	100000
class.mean.diff[1,6]	-8.7	1.541	0.02275	-11.78	-8.686	-5.747	50001	100000
class.mean.diff[1,7]	-5.758	1.254	0.02213	-8.232	-5.744	-3.314	50001	100000
class.mean.diff[1,8]	-0.103	2.164	0.02634	-4.344	-0.1013	4.112	50001	100000
class.mean.diff[1,9]	-10.67	1.916	0.02438	-14.42	-10.68	-6.9	50001	100000
class.mean.diff[1,10]	-0.9254	1.563	0.02449	-4.097	-0.8947	2.087	50001	100000

Note: Reference class appears first, for example class.mean.diff[1,3] shows the mean reduction in YBOCS of SSRIs vs placebo

Individual Effects vs. Placebo (see key for individual interventions)

	**mean**	**sd**	**MC_error**	**val2.5pc**	**median**	**val97.5pc**	**start**	**sample**
treat.mean.diff[1,2]	2.063	1.806	0.03318	-1.509	2.067	5.611	50001	100000
treat.mean.diff[1,3]	-3.374	0.4854	0.007081	-4.367	-3.369	-2.426	50001	100000
treat.mean.diff[1,4]	-3.44	0.4873	0.007192	-4.479	-3.422	-2.535	50001	100000
treat.mean.diff[1,5]	-3.038	0.4909	0.007551	-3.924	-3.067	-1.993	50001	100000

treat.mean.diff[1,6]	-3.486	0.5394	0.007976	-4.662	-3.455	-2.499	50001	100000
treat.mean.diff[1,7]	-3.366	0.5748	0.006907	-4.579	-3.357	-2.24	50001	100000
treat.mean.diff[1,8]	-2.458	1.533	0.01317	-5.492	-2.461	0.5665	50001	100000
treat.mean.diff[1,9]	-3.156	0.6228	0.008235	-4.392	-3.151	-1.95	50001	100000
treat.mean.diff[1,10]	-8.7	1.541	0.02275	-11.78	-8.686	-5.747	50001	100000
treat.mean.diff[1,11]	-5.758	1.254	0.02213	-8.232	-5.744	-3.314	50001	100000
treat.mean.diff[1,12]	-0.103	2.164	0.02634	-4.344	-0.1013	4.112	50001	100000
treat.mean.diff[1,13]	-10.67	1.916	0.02438	-14.42	-10.68	-6.9	50001	100000
treat.mean.diff[1,14]	-3.289	0.583	0.007209	-4.447	-3.3	-2.07	50001	100000
treat.mean.diff[1,15]	-0.9254	1.563	0.02449	-4.097	-0.8947	2.087	50001	100000

Note: Reference class appears first, for example treat.mean.diff[1,3] shows the mean reduction in YBOCS of fluoxetine vs placebo

B2.3 Detailed Results of the Sensitivity Analysis 3 (Blinding)

Raw Data Used

Studyid	y[,1]	n[,1]	sd[,1]	y[,2]	n[,2]	sd[,2]	y[,3]	n[,3]	sd[,3]	y[,4]	n[,4]	sd[,4]	na[]	out[]	t[,1]	t[,2]	t[,3]	t[,4]
#Albert2001	18.36	25	7.11	17.3	40	6.15	NA	NA	NA	NA	NA	NA	2	2	8	9	NA	NA
#Andersson2012	12.94	49	6.26	18.88	51	4.18	NA	NA	NA	NA	NA	NA	2	2	11	15	NA	NA
#Belloch2008	8.31	13	8.75	6.8	16	3.55	NA	NA	NA	NA	NA	NA	2	2	10	12	NA	NA
#Belotto-Silva2012	20.29	88	8.05	19.97	70	8.48	NA	NA	NA	NA	NA	NA	2	2	3	11	NA	NA
#Cordioli2003	23.2	24	5.5	15.1	23	7.8	NA	NA	NA	NA	NA	NA	2	2	2	11	NA	NA
#Cottraux2001	-12.1	30	7.8	-12.5	30	8.2	NA	NA	NA	NA	NA	NA	2	1	10	12	NA	NA
#Denys2003	-7.8	72	5.4	-7.2	73	7.5	NA	NA	NA	NA	NA	NA	2	1	5	8	NA	NA
#Foa2005	22.2	26	6.4	18.2	36	7.8	11	29	7.9	10.5	31	8.2	4	2	1	9	10	13
#Goodman1989	28	21	7	19.4	21	7	NA	NA	NA	NA	NA	NA	2	2	1	4	NA	NA
#Jenike1990b	21.8	20	7.6	18.8	18	4	NA	NA	NA	NA	NA	NA	2	2	1	4	NA	NA
#Mundo1997	16.2	10	8.9	21.6	9	7.6	19.8	11	10.1	NA	NA	NA	3	2	4	5	7	NA
#Nakatani2005	20.2	10	9.4	12.9	10	4.9	28.4	8	5.5	NA	NA	NA	3	2	4	10	15	NA
#O'Connor2006	25.4	10	3.5	24	11	4.7	NA	NA	NA	NA	NA	NA	2	2	1	4	NA	NA
#O'Konnor1999	17.5	6	4	13.3	6	8.6	NA	NA	NA	NA	NA	NA	2	2	2	11	NA	NA
#Stein2007	-8.46	113	8.08	-11.67	116	8.40	-11.43	112	8.25	-12.14	114	8.22	4	1	1	5	14	14
#Whittal2005	10.41	29	7.6	10.6	30	7.1	NA	NA	NA	NA	NA	NA	2	2	10	12	NA	NA
#Sousa2006	-7.36	25	NA	-10.8	25	NA	NA	NA	NA	NA	NA	NA	2	1	6	11	NA	NA

Key

1 Placebo (1)
2 Waitlist (2)
3 Fluoxetine (3)

4 Fluvoxamine (3)
5 Paroxetine (3)
6 Sertraline (3)
7 Citalopram (3)
8 Venlafaxine (4)
9 Clomipramine (5)
10 Behavioural Therapy [BT] (6)
11 CBT (7)
12 Cognitive Therapy (8)
13 BT + Clomipramine (9)
14 Escitalopram (3)
15 Psychological Placebo (10)

Class Effects vs. Placebo (see key for class interventions)

	mean	sd	MC_error	val2.5pc	median	val97.5pc	start	sample
class.mean.diff[1,2]	3.235	2.714	0.06924	-2.16	3.238	8.44	50001	100000
class.mean.diff[1,3]	-3.305	1.234	0.02794	-5.59	-3.349	-0.6521	50001	100000
class.mean.diff[1,4]	-2.728	1.632	0.0314	-5.968	-2.7	0.4702	50001	100000
class.mean.diff[1,5]	-4.046	1.674	0.03403	-7.303	-4.023	-0.73	50001	100000
class.mean.diff[1,6]	-11.79	1.762	0.04753	-15.17	-11.81	-8.279	50001	100000
class.mean.diff[1,7]	-4.112	1.842	0.04886	-7.631	-4.125	-0.3389	50001	100000
class.mean.diff[1,8]	-12.23	2.231	0.05662	-16.66	-12.24	-7.796	50001	100000
class.mean.diff[1,9]	-11.85	2.064	0.03817	-16.07	-11.85	-7.821	50001	100000
class.mean.diff[1,10]	2.328	1.991	0.0483	-1.486	2.28	6.364	50001	100000

Note: Reference class appears first, for example class.mean.diff[1,3] shows the mean reduction in YBOCS of SSRIs vs placebo

Individual Effects vs. Placebo (see key for individual interventions)

	mean	sd	MC_error	val2.5pc	median	val97.5pc	start	sample
treat.mean.diff[1,2]	3.235	2.714	0.06924	-2.16	3.238	8.44	50001	100000
treat.mean.diff[1,3]	-3.416	1.611	0.03769	-6.618	-3.456	0.06601	50001	100000
treat.mean.diff[1,4]	-3.849	1.018	0.02311	-5.988	-3.813	-1.948	50001	100000
treat.mean.diff[1,5]	-3.203	1.103	0.02543	-5.291	-3.238	-0.8988	50001	100000
treat.mean.diff[1,6]	-2.713	1.897	0.04831	-5.647	-3.041	2.035	50001	100000
treat.mean.diff[1,7]	-3.272	1.64	0.03575	-6.501	-3.333	0.4173	50001	100000
treat.mean.diff[1,8]	-2.728	1.632	0.0314	-5.968	-2.7	0.4702	50001	100000
treat.mean.diff[1,9]	-4.046	1.674	0.03403	-7.303	-4.023	-0.73	50001	100000
treat.mean.diff[1,10]	-11.79	1.762	0.04753	-15.17	-11.81	-8.279	50001	100000
treat.mean.diff[1,11]	-4.112	1.842	0.04886	-7.631	-4.125	-0.3389	50001	100000
treat.mean.diff[1,12]	-12.23	2.231	0.05662	-16.66	-12.24	-7.796	50001	100000
treat.mean.diff[1,13]	-11.85	2.064	0.03817	-16.07	-11.85	-7.821	50001	100000
treat.mean.diff[1,14]	-3.356	1.058	0.02341	-5.376	-3.374	-1.212	50001	100000
treat.mean.diff[1,15]	2.328	1.991	0.0483	-1.486	2.28	6.364	50001	100000

Note: Reference class appears first, for example treat.mean.diff[1,3] shows the mean reduction in YBOCS of fluoxetine vs placebo

B3. Detailed Results of the secondary analysis (excluding waiting list controlled trials) presented in tables 2 and 3 (right columns) of the manuscript

KEY

Intervention (Class)

1 PLA (1)
2 FLX (2)
3 FLV (2)
4 PAR (2)
5 SER (2)
6 CIT (2)
7 VEN (3)
8 CLO (4)
9 BT (5)
10 CBT (6)
11 CT (7)
12 Hypericum (8)
13 FLV+CBT (9)
14 BT + CLO (10)
15 s-CIT (2)
16 PsychPLA (11)

Class Effects vs. Placebo (see key for class interventions)

	mean	sd	MC_error	val2.5pc	median	val97.5pc	start	sample
class.mean.diff[1,2]	-3.619	0.6562	0.008122	-4.889	-3.616	-2.341	72201	140000
class.mean.diff[1,3]	-3.211	1.965	0.01096	-7.068	-3.222	0.6873	72201	140000

class.mean.diff[1,4]	-4.665	0.8162	0.00625	-6.264	-4.668	-3.046	72201	140000
class.mean.diff[1,5]	-10.41	1.854	0.02225	-14.04	-10.41	-6.774	72201	140000
class.mean.diff[1,6]	-7.981	1.544	0.01264	-11.02	-7.982	-4.93	72201	140000
class.mean.diff[1,7]	-9.452	2.179	0.02475	-13.76	-9.447	-5.195	72201	140000
class.mean.diff[1,8]	-0.1281	2.957	0.01417	-5.932	-0.125	5.678	72201	140000
class.mean.diff[1,9]	-8.808	2.5	0.01532	-13.75	-8.8	-3.879	72201	140000
class.mean.diff[1,10]	-11.68	2.565	0.01434	-16.73	-11.68	-6.655	72201	140000
class.mean.diff[1,11]	-1.896	1.923	0.02168	-5.621	-1.901	1.908	72201	140000
class.mean.diff[2,3]	0.4075	1.951	0.008553	-3.417	0.407	4.26	72201	140000
class.mean.diff[2,4]	-1.046	0.8537	0.004422	-2.728	-1.045	0.6274	72201	140000
class.mean.diff[2,5]	-6.791	1.869	0.02092	-10.44	-6.796	-3.11	72201	140000
class.mean.diff[2,6]	-4.362	1.506	0.009316	-7.338	-4.359	-1.404	72201	140000
class.mean.diff[2,7]	-5.834	2.192	0.02333	-10.17	-5.826	-1.515	72201	140000
class.mean.diff[2,8]	3.491	3.026	0.01591	-2.45	3.494	9.439	72201	140000
class.mean.diff[2,9]	-5.189	2.477	0.01263	-10.09	-5.179	-0.3343	72201	140000
class.mean.diff[2,10]	-8.065	2.604	0.0139	-13.18	-8.061	-2.953	72201	140000
class.mean.diff[2,11]	1.723	1.926	0.02002	-2.015	1.721	5.544	72201	140000
class.mean.diff[3,4]	-1.454	1.927	0.008479	-5.254	-1.45	2.334	72201	140000
class.mean.diff[3,5]	-7.198	2.632	0.02236	-12.38	-7.204	-2.048	72201	140000
class.mean.diff[3,6]	-4.77	2.421	0.01284	-9.527	-4.771	0.005828	72201	140000
class.mean.diff[3,7]	-6.241	2.87	0.02466	-11.89	-6.232	-0.6171	72201	140000
class.mean.diff[3,8]	3.083	3.54	0.01781	-3.913	3.075	10.05	72201	140000
class.mean.diff[3,9]	-5.597	3.119	0.01543	-11.78	-5.59	0.4985	72201	140000
class.mean.diff[3,10]	-8.472	3.166	0.01546	-14.7	-8.483	-2.256	72201	140000
class.mean.diff[3,11]	1.316	2.678	0.0217	-3.927	1.303	6.6	72201	140000
class.mean.diff[4,5]	-5.745	1.894	0.02046	-9.462	-5.749	-2.025	72201	140000
class.mean.diff[4,6]	-3.316	1.629	0.00976	-6.53	-3.31	-0.1128	72201	140000
class.mean.diff[4,7]	-4.788	2.219	0.02298	-9.16	-4.776	-0.4485	72201	140000
class.mean.diff[4,8]	4.537	3.063	0.01532	-1.482	4.536	10.56	72201	140000

class.mean.diff[4,9]	-4.143	2.555	0.01295	-9.193	-4.134	0.8686	72201	140000
class.mean.diff[4,10]	-7.019	2.576	0.0129	-12.1	-7.018	-1.976	72201	140000
class.mean.diff[4,11]	2.769	1.97	0.01966	-1.083	2.764	6.666	72201	140000
class.mean.diff[5,6]	2.429	1.975	0.01597	-1.481	2.434	6.315	72201	140000
class.mean.diff[5,7]	0.9571	1.257	0.006965	-1.519	0.9601	3.408	72201	140000
class.mean.diff[5,8]	10.28	3.491	0.02624	3.401	10.27	17.14	72201	140000
class.mean.diff[5,9]	1.602	2.9	0.02016	-4.127	1.611	7.287	72201	140000
class.mean.diff[5,10]	-1.274	2.764	0.01683	-6.711	-1.272	4.133	72201	140000
class.mean.diff[5,11]	8.514	1.258	0.005157	6.079	8.502	11.04	72201	140000
class.mean.diff[6,7]	-1.472	2.26	0.01843	-5.918	-1.467	2.978	72201	140000
class.mean.diff[6,8]	7.853	3.334	0.01882	1.305	7.855	14.4	72201	140000
class.mean.diff[6,9]	-0.827	2.403	0.008894	-5.572	-0.813	3.882	72201	140000
class.mean.diff[6,10]	-3.703	2.868	0.01335	-9.372	-3.712	1.936	72201	140000
class.mean.diff[6,11]	6.085	1.898	0.01477	2.389	6.067	9.874	72201	140000
class.mean.diff[7,8]	9.324	3.675	0.02848	2.094	9.313	16.57	72201	140000
class.mean.diff[7,9]	0.6445	3.109	0.02217	-5.482	0.649	6.764	72201	140000
class.mean.diff[7,10]	-2.231	3.008	0.01898	-8.162	-2.224	3.676	72201	140000
class.mean.diff[7,11]	7.557	1.581	0.008246	4.492	7.544	10.7	72201	140000
class.mean.diff[8,9]	-8.68	3.868	0.0205	-16.32	-8.66	-1.088	72201	140000
class.mean.diff[8,10]	-11.56	3.923	0.02016	-19.28	-11.54	-3.879	72201	140000
class.mean.diff[8,11]	-1.767	3.529	0.02581	-8.669	-1.774	5.208	72201	140000
class.mean.diff[9,10]	-2.876	3.509	0.01675	-9.797	-2.892	4.067	72201	140000
class.mean.diff[9,11]	6.912	2.894	0.01907	1.262	6.891	12.69	72201	140000
class.mean.diff[10,11]	9.788	2.884	0.01663	4.167	9.764	15.49	72201	140000

Individual Effects vs. Placebo (see key for individual interventions)

	mean	sd	MC_error	val2.5pc	median	val97.5pc	start	sample
treat.mean.diff[1,2]	-3.668	0.7231	0.008129	-5.126	-3.655	-2.26	72201	140000
treat.mean.diff[1,3]	-3.658	0.6577	0.007807	-4.964	-3.648	-2.375	72201	140000
treat.mean.diff[1,4]	-3.513	0.6774	0.007931	-4.809	-3.528	-2.137	72201	140000
treat.mean.diff[1,5]	-3.684	0.717	0.007973	-5.141	-3.67	-2.296	72201	140000
treat.mean.diff[1,6]	-3.602	0.83	0.008281	-5.251	-3.606	-1.906	72201	140000
treat.mean.diff[1,7]	-3.211	1.965	0.01096	-7.068	-3.222	0.6873	72201	140000
treat.mean.diff[1,8]	-4.665	0.8162	0.00625	-6.264	-4.668	-3.046	72201	140000
treat.mean.diff[1,9]	-10.41	1.854	0.02225	-14.04	-10.41	-6.774	72201	140000
treat.mean.diff[1,10]	-7.981	1.544	0.01264	-11.02	-7.982	-4.93	72201	140000
treat.mean.diff[1,11]	-9.452	2.179	0.02475	-13.76	-9.447	-5.195	72201	140000
treat.mean.diff[1,12]	-0.1281	2.957	0.01417	-5.932	-0.125	5.678	72201	140000
treat.mean.diff[1,13]	-8.808	2.5	0.01532	-13.75	-8.8	-3.879	72201	140000
treat.mean.diff[1,14]	-11.68	2.565	0.01434	-16.73	-11.68	-6.655	72201	140000
treat.mean.diff[1,15]	-3.593	0.8384	0.008388	-5.247	-3.598	-1.861	72201	140000
treat.mean.diff[1,16]	-1.896	1.923	0.02168	-5.621	-1.901	1.908	72201	140000
treat.mean.diff[2,3]	0.01052	0.6833	0.002766	-1.467	0.002346	1.521	72201	140000
treat.mean.diff[2,4]	0.1557	0.7075	0.003288	-1.211	0.05288	1.846	72201	140000
treat.mean.diff[2,5]	-0.01618	0.6854	0.002483	-1.512	-0.004387	1.468	72201	140000
treat.mean.diff[2,6]	0.06622	0.8189	0.003145	-1.632	0.01439	1.943	72201	140000
treat.mean.diff[2,7]	0.457	1.992	0.00902	-3.458	0.4529	4.404	72201	140000
treat.mean.diff[2,8]	-0.9965	0.9169	0.00464	-2.789	-1.005	0.846	72201	140000
treat.mean.diff[2,9]	-6.741	1.886	0.02071	-10.44	-6.744	-3.024	72201	140000
treat.mean.diff[2,10]	-4.313	1.502	0.009203	-7.28	-4.313	-1.354	72201	140000
treat.mean.diff[2,11]	-5.784	2.205	0.02311	-10.13	-5.781	-1.462	72201	140000

treat.mean.diff[2,12]	3.54	3.041	0.01597	-2.428	3.54	9.522	72201	140000
treat.mean.diff[2,13]	-5.14	2.492	0.01266	-10.07	-5.127	-0.2542	72201	140000
treat.mean.diff[2,14]	-8.015	2.62	0.01378	-13.17	-8.013	-2.882	72201	140000
treat.mean.diff[2,15]	0.07572	0.8179	0.002934	-1.595	0.0168	1.995	72201	140000
treat.mean.diff[2,16]	1.773	1.937	0.01981	-1.992	1.764	5.617	72201	140000
treat.mean.diff[3,4]	0.1451	0.6511	0.003189	-1.116	0.05422	1.686	72201	140000
treat.mean.diff[3,5]	-0.0267	0.6763	0.002749	-1.534	-0.005748	1.42	72201	140000
treat.mean.diff[3,6]	0.05569	0.7824	0.003341	-1.585	0.01283	1.837	72201	140000
treat.mean.diff[3,7]	0.4465	1.967	0.008816	-3.408	0.4423	4.341	72201	140000
treat.mean.diff[3,8]	-1.007	0.8361	0.00416	-2.637	-1.011	0.6582	72201	140000
treat.mean.diff[3,9]	-6.752	1.848	0.02079	-10.36	-6.763	-3.101	72201	140000
treat.mean.diff[3,10]	-4.323	1.492	0.009301	-7.249	-4.326	-1.386	72201	140000
treat.mean.diff[3,11]	-5.795	2.175	0.02314	-10.08	-5.791	-1.512	72201	140000
treat.mean.diff[3,12]	3.53	3.027	0.01573	-2.408	3.528	9.494	72201	140000
treat.mean.diff[3,13]	-5.15	2.444	0.01241	-9.968	-5.144	-0.3437	72201	140000
treat.mean.diff[3,14]	-8.026	2.598	0.01376	-13.14	-8.025	-2.942	72201	140000
treat.mean.diff[3,15]	0.0652	0.7871	0.003045	-1.558	0.0142	1.873	72201	140000
treat.mean.diff[3,16]	1.762	1.906	0.01996	-1.931	1.757	5.542	72201	140000
treat.mean.diff[4,5]	-0.1718	0.7048	0.00316	-1.864	-0.06107	1.166	72201	140000
treat.mean.diff[4,6]	-0.08944	0.7842	0.003025	-1.878	-0.02549	1.525	72201	140000
treat.mean.diff[4,7]	0.3014	1.903	0.008076	-3.445	0.2969	4.052	72201	140000
treat.mean.diff[4,8]	-1.152	0.8443	0.004138	-2.832	-1.143	0.5	72201	140000
treat.mean.diff[4,9]	-6.897	1.889	0.02099	-10.59	-6.902	-3.195	72201	140000
treat.mean.diff[4,10]	-4.468	1.548	0.009658	-7.544	-4.463	-1.427	72201	140000
treat.mean.diff[4,11]	-5.94	2.208	0.02342	-10.29	-5.932	-1.6	72201	140000
treat.mean.diff[4,12]	3.384	3.033	0.01579	-2.586	3.381	9.337	72201	140000
treat.mean.diff[4,13]	-5.295	2.502	0.01292	-10.25	-5.28	-0.4009	72201	140000
treat.mean.diff[4,14]	-8.171	2.608	0.01393	-13.3	-8.167	-3.061	72201	140000

treat.mean.diff[4,15]	-0.07994	0.7558	0.002627	-1.823	-0.02358	1.494	72201	140000
treat.mean.diff[4,16]	1.617	1.947	0.02014	-2.17	1.611	5.485	72201	140000
treat.mean.diff[5,6]	0.0824	0.8161	0.003144	-1.588	0.01993	1.97	72201	140000
treat.mean.diff[5,7]	0.4732	1.985	0.008831	-3.435	0.4678	4.412	72201	140000
treat.mean.diff[5,8]	-0.9803	0.9039	0.004381	-2.749	-0.9886	0.8367	72201	140000
treat.mean.diff[5,9]	-6.725	1.891	0.02078	-10.43	-6.728	-2.999	72201	140000
treat.mean.diff[5,10]	-4.296	1.516	0.00936	-7.283	-4.3	-1.317	72201	140000
treat.mean.diff[5,11]	-5.768	2.211	0.02321	-10.14	-5.768	-1.412	72201	140000
treat.mean.diff[5,12]	3.556	3.038	0.01588	-2.415	3.556	9.524	72201	140000
treat.mean.diff[5,13]	-5.123	2.494	0.01267	-10.04	-5.117	-0.2244	72201	140000
treat.mean.diff[5,14]	-7.999	2.619	0.01382	-13.14	-8.001	-2.874	72201	140000
treat.mean.diff[5,15]	0.0919	0.8153	0.002986	-1.567	0.02133	1.997	72201	140000
treat.mean.diff[5,16]	1.789	1.945	0.01995	-1.98	1.778	5.658	72201	140000
treat.mean.diff[6,7]	0.3908	2.026	0.008788	-3.607	0.3908	4.402	72201	140000
treat.mean.diff[6,8]	-1.063	1.009	0.004924	-3.089	-1.059	0.9414	72201	140000
treat.mean.diff[6,9]	-6.807	1.946	0.02113	-10.64	-6.81	-2.969	72201	140000
treat.mean.diff[6,10]	-4.379	1.606	0.009639	-7.578	-4.373	-1.233	72201	140000
treat.mean.diff[6,11]	-5.85	2.258	0.02351	-10.32	-5.846	-1.416	72201	140000
treat.mean.diff[6,12]	3.474	3.068	0.01582	-2.554	3.477	9.493	72201	140000
treat.mean.diff[6,13]	-5.206	2.536	0.01286	-10.25	-5.188	-0.2367	72201	140000
treat.mean.diff[6,14]	-8.081	2.662	0.01417	-13.32	-8.077	-2.856	72201	140000
treat.mean.diff[6,15]	0.009508	0.8838	0.002996	-1.894	0.001128	1.97	72201	140000
treat.mean.diff[6,16]	1.706	2.002	0.02021	-2.191	1.709	5.673	72201	140000
treat.mean.diff[7,8]	-1.454	1.927	0.008479	-5.254	-1.45	2.334	72201	140000
treat.mean.diff[7,9]	-7.198	2.632	0.02236	-12.38	-7.204	-2.048	72201	140000
treat.mean.diff[7,10]	-4.77	2.421	0.01284	-9.527	-4.771	0.005828	72201	140000
treat.mean.diff[7,11]	-6.241	2.87	0.02466	-11.89	-6.232	-0.6171	72201	140000
treat.mean.diff[7,12]	3.083	3.54	0.01781	-3.913	3.075	10.05	72201	140000
treat.mean.diff[7,13]	-5.597	3.119	0.01543	-11.78	-5.59	0.4985	72201	140000

treat.mean.diff[7,14]	-8.472	3.166	0.01546	-14.7	-8.483	-2.256	72201	140000
treat.mean.diff[7,15]	-0.3813	2.018	0.008616	-4.374	-0.3787	3.581	72201	140000
treat.mean.diff[7,16]	1.316	2.678	0.0217	-3.927	1.303	6.6	72201	140000
treat.mean.diff[8,9]	-5.745	1.894	0.02046	-9.462	-5.749	-2.025	72201	140000
treat.mean.diff[8,10]	-3.316	1.629	0.00976	-6.53	-3.31	-0.1128	72201	140000
treat.mean.diff[8,11]	-4.788	2.219	0.02298	-9.16	-4.776	-0.4485	72201	140000
treat.mean.diff[8,12]	4.537	3.063	0.01532	-1.482	4.536	10.56	72201	140000
treat.mean.diff[8,13]	-4.143	2.555	0.01295	-9.193	-4.134	0.8686	72201	140000
treat.mean.diff[8,14]	-7.019	2.576	0.0129	-12.1	-7.018	-1.976	72201	140000
treat.mean.diff[8,15]	1.072	1.009	0.004804	-0.9283	1.068	3.095	72201	140000
treat.mean.diff[8,16]	2.769	1.97	0.01966	-1.083	2.764	6.666	72201	140000
treat.mean.diff[9,10]	2.429	1.975	0.01597	-1.481	2.434	6.315	72201	140000
treat.mean.diff[9,11]	0.9571	1.257	0.006965	-1.519	0.9601	3.408	72201	140000
treat.mean.diff[9,12]	10.28	3.491	0.02624	3.401	10.27	17.14	72201	140000
treat.mean.diff[9,13]	1.602	2.9	0.02016	-4.127	1.611	7.287	72201	140000
treat.mean.diff[9,14]	-1.274	2.764	0.01683	-6.711	-1.272	4.133	72201	140000
treat.mean.diff[9,15]	6.817	1.948	0.02106	2.984	6.817	10.64	72201	140000
treat.mean.diff[9,16]	8.514	1.258	0.005157	6.079	8.502	11.04	72201	140000
treat.mean.diff[10,11]	-1.472	2.26	0.01843	-5.918	-1.467	2.978	72201	140000
treat.mean.diff[10,12]	7.853	3.334	0.01882	1.305	7.855	14.4	72201	140000
treat.mean.diff[10,13]	-0.827	2.403	0.008894	-5.572	-0.813	3.882	72201	140000
treat.mean.diff[10,14]	-3.703	2.868	0.01335	-9.372	-3.712	1.936	72201	140000
treat.mean.diff[10,15]	4.388	1.611	0.009541	1.232	4.381	7.596	72201	140000
treat.mean.diff[10,16]	6.085	1.898	0.01477	2.389	6.067	9.874	72201	140000
treat.mean.diff[11,12]	9.324	3.675	0.02848	2.094	9.313	16.57	72201	140000
treat.mean.diff[11,13]	0.6445	3.109	0.02217	-5.482	0.649	6.764	72201	140000
treat.mean.diff[11,14]	-2.231	3.008	0.01898	-8.162	-2.224	3.676	72201	140000
treat.mean.diff[11,15]	5.86	2.259	0.02345	1.426	5.857	10.33	72201	140000
treat.mean.diff[11,16]	7.557	1.581	0.008246	4.492	7.544	10.7	72201	140000

treat.mean.diff[12,13]	-8.68	3.868	0.0205	-16.32	-8.66	-1.088	72201	140000
treat.mean.diff[12,14]	-11.56	3.923	0.02016	-19.28	-11.54	-3.879	72201	140000
treat.mean.diff[12,15]	-3.464	3.066	0.01607	-9.484	-3.474	2.576	72201	140000
treat.mean.diff[12,16]	-1.767	3.529	0.02581	-8.669	-1.774	5.208	72201	140000
treat.mean.diff[13,14]	-2.876	3.509	0.01675	-9.797	-2.892	4.067	72201	140000
treat.mean.diff[13,15]	5.215	2.539	0.013	0.2461	5.2	10.24	72201	140000
treat.mean.diff[13,16]	6.912	2.894	0.01907	1.262	6.891	12.69	72201	140000
treat.mean.diff[14,15]	8.091	2.659	0.01413	2.899	8.08	13.34	72201	140000
treat.mean.diff[14,16]	9.788	2.884	0.01663	4.167	9.764	15.49	72201	140000
treat.mean.diff[15,16]	1.697	2.004	0.02016	-2.224	1.696	5.665	72201	140000

Figure A1: Risk of Bias assessment

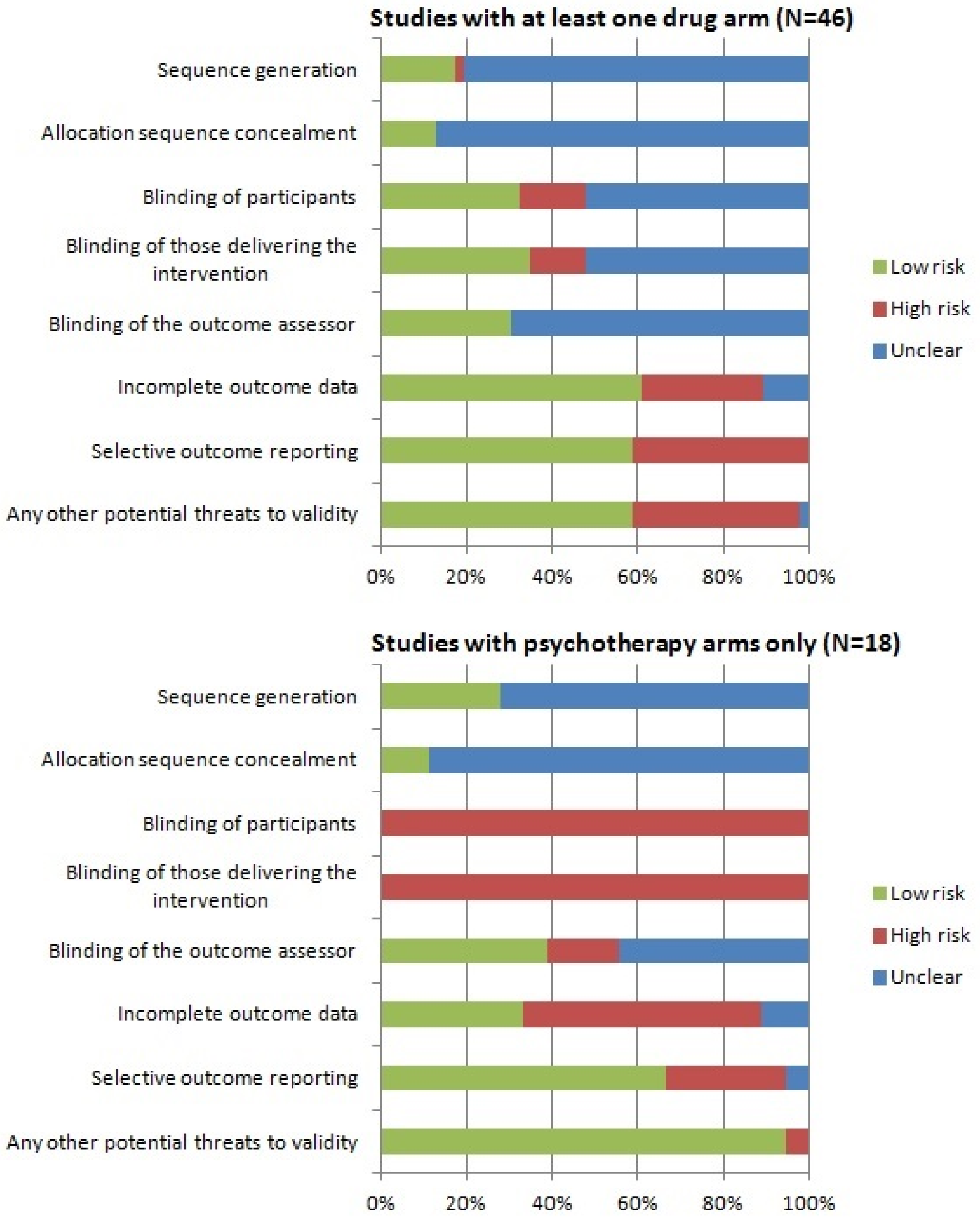

Figure A2: Rankograms - blue line: efficacy (mean difference in YBOCS); orange line: acceptability (total dropout rates)

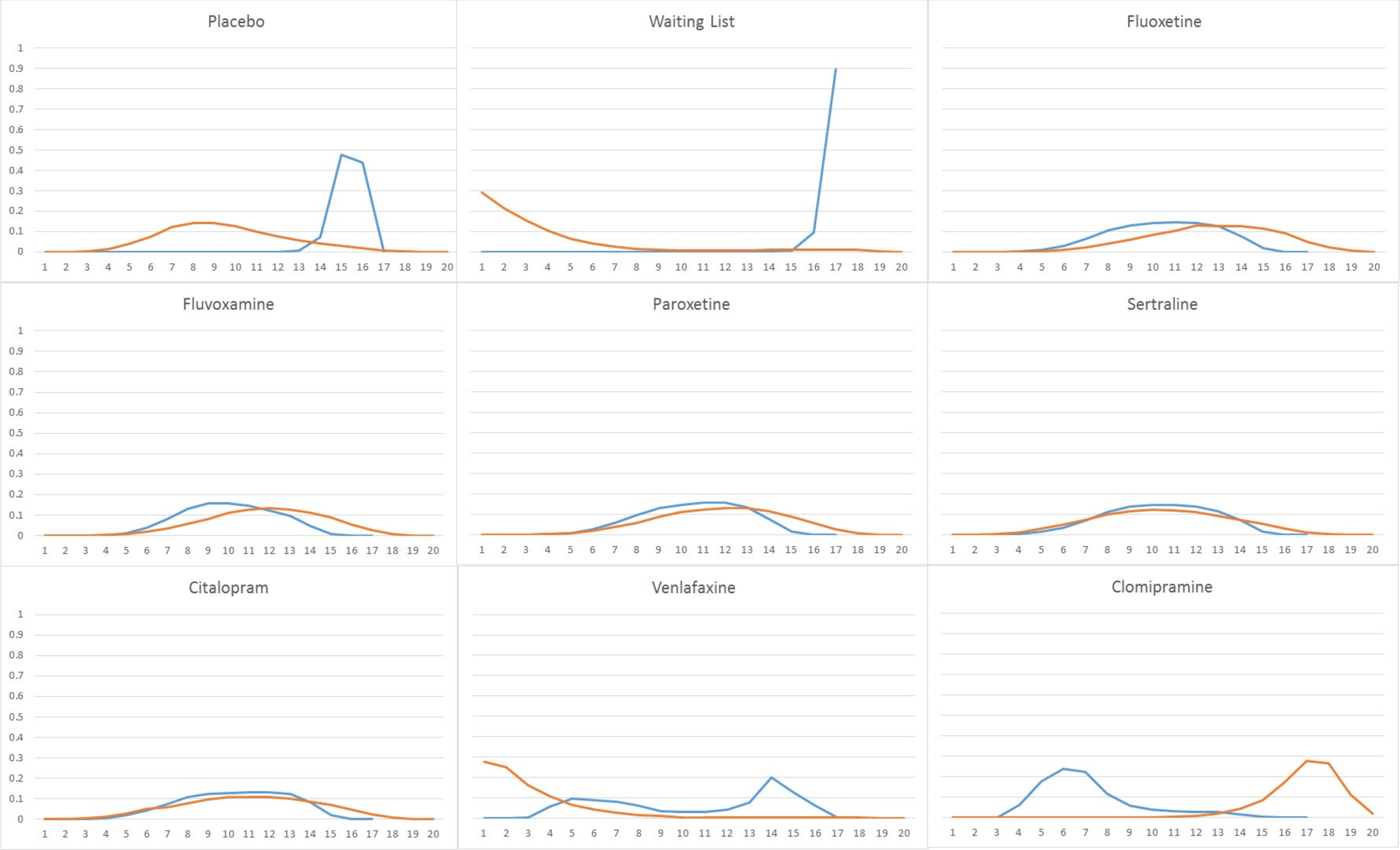

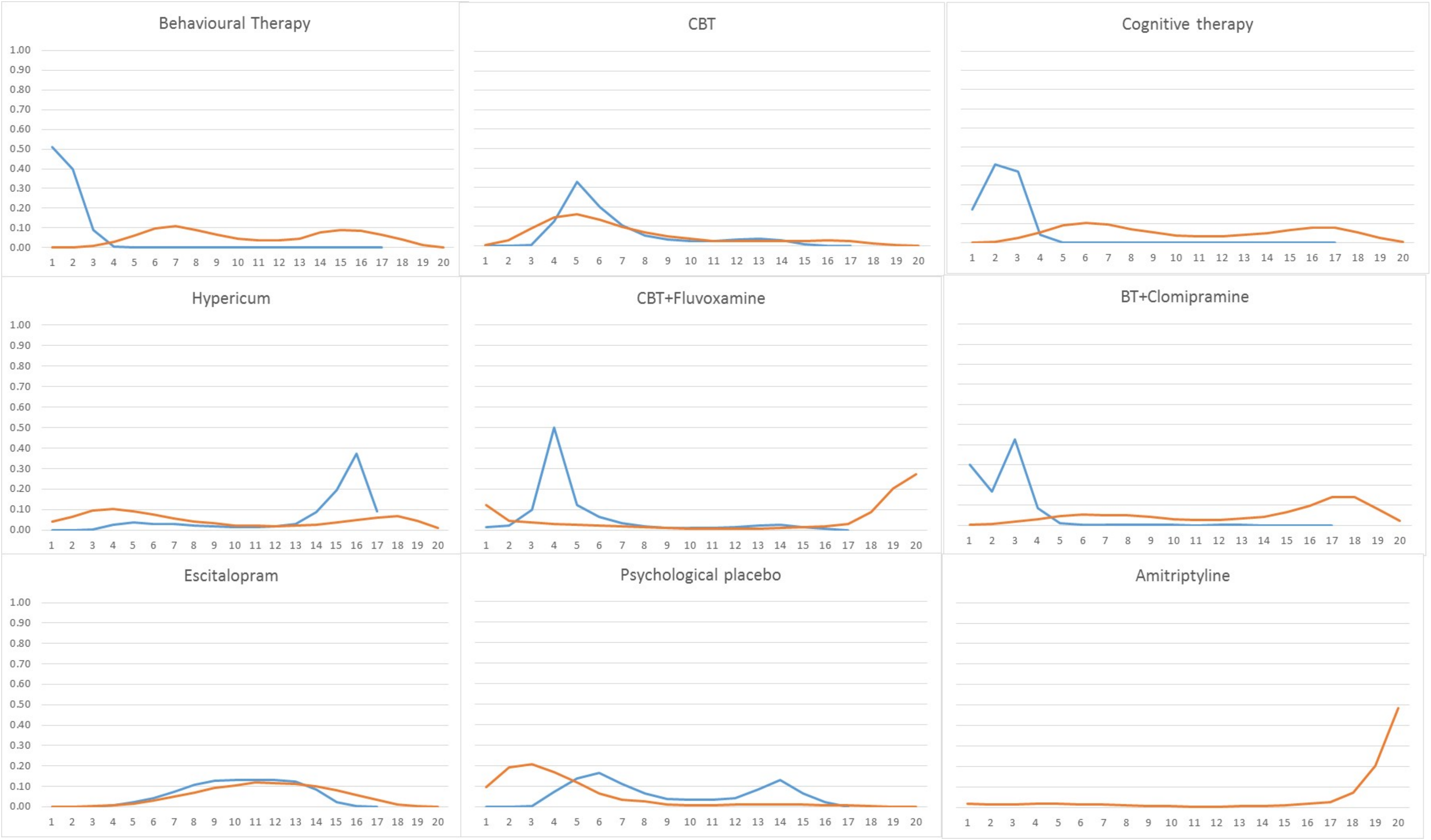
Behavioural Therapy
CBT
Cognitive therapy
Hypericum
CBT+Fluvoxamine
BT+Clomipramine
Escitalopram
Psychological placebo
Amitriptyline
1.00
0.90
0.80
0.70
0.60
0.50
0.40
0.30
0.20
0.10
0.00
1 2 3 4 5 6 7 8 9 10 11 12 13 14 15 16 17 18 19 20

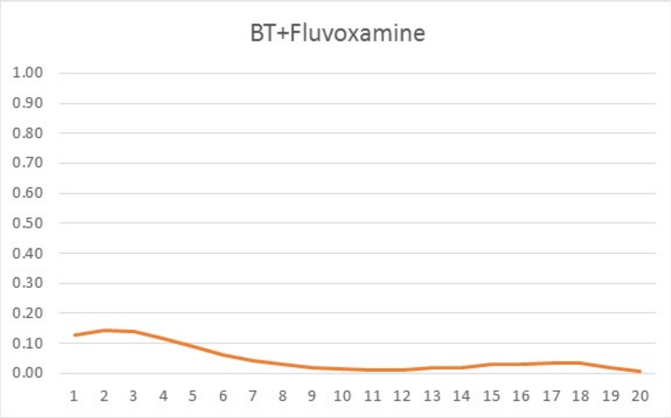
BT+Fluvoxamine
1.00
0.90
0.80
0.70
0.60
0.50
0.40
0.30
0.20
0.10
0.00
1 2 3 4 5 6 7 8 9 10 11 12 13 14 15 16 17 18 19 20

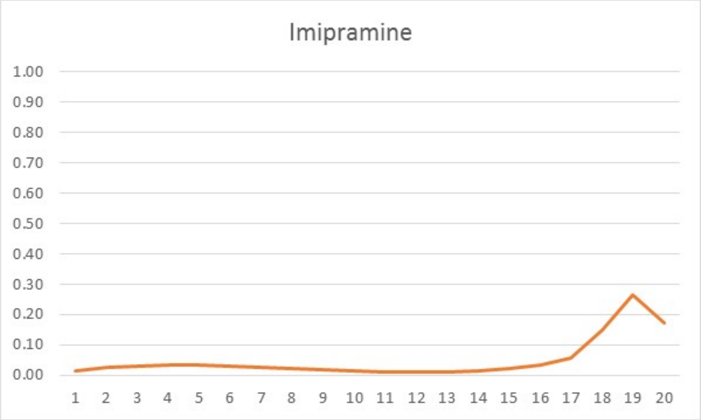
Imipramine
1.00
0.90
0.80
0.70
0.60
0.50
0.40
0.30
0.20
0.10
0.00
1 2 3 4 5 6 7 8 9 10 11 12 13 14 15 16 17 18 19 20

Table A1 Detailed description of 64 trials included in the Systematic Review. Studies (n=54) included in the quantitative synthesis (network meta-analysis) are shown in bold

StudyID	N of patients	N of Arms	Intervention type	Arm1	Arm2	Arm3	Arm4	Duration (weeks)	Mean Age	% female	Scale	Comorbid Depression	Industry Sponsored *	Allowed concurrent antidepressant use (% of patients on drugs) **
**Albert2001**[22]	73	2	drug	VEN	CLO	-	-	12	29·65	47·9	YBOCS	No	No	-
Ananth1981[23]	20	2	drug	CLO	AMI	-	-	4	36·9	65	Other	Yes	Unclear	-
**Anderson2007**[24]	38	2	therapy	CBT	WL	-	-	10	33·18	-	YBOCS	Yes	-	Yes (65% Arm1, 57% Arm2)
**Andersson2012**[25]	101	2	therapy	CBT	PsyPLA	-	-	10	34	66	YBOCS	Yes	-	Yes (23% total)
**Belloch2008**[26]	33	2	therapy	BT	CT	-	-	24	32	-	YBOCS	Yes	-	Yes (62% total)
**Belotto-Silva2012**[27]	158	2	combination	FLX	CBT	-	-	12	34·04	55	YBOCS	Yes	No	-
**Bergeron2002**[28]	150	2	drug	FLX	SER	-	-	24	36·53	54	YBOCS	No	Yes	-
**Bisserbe1997**[29]	168	2	drug	SER	CLO	-	-	16	39·77	63	YBOCS	No	Yes	-
**Chouinard1990** [30]	87	2	drug	SER	PLA	-	-	8	37·25	15	YBOCS	No	Yes	-
**CCSG11991** [31]	239	2	drug	CLO	PLA	-	-	10	35·4	61	YBOCS	No	Yes	-
**CCSG21991** [31]	281	2	drug	CLO	PLA	-	-	10	35·6	51·5	YBOCS	No	Yes	-
**Cordioli2003** [32]	47	2	therapy	CBT	WL	-	-	12	36·5	51	YBOCS	Yes	-	Yes (45% total)
Cottraux1993[33]	60	3	combination	FLV	BT	BT+ FLV	-	24	-	63	Other	Yes	No	-
**Cottraux2001**[34]	65	2	therapy	BT	CT	-	-	16	35·78	74	YBOCS	No	-	Yes (hypnotic drugs by design, but 13% at the end of study on antidepressant)
**Denys2003**[35]	150	2	drug	PAR	VEN	-	-	12	35	62	YBOCS	No	Yes	-
Emmelkamp1988[36]	20	2	therapy	CT	BT	-	-	8	29·9	-	Other	Yes	-	No (by design, at the end 3 reported use)
Emmelkamp1991[37]	30	2	therapy	CT	BT	-	-	4	-	76	Other	Yes	-	No (by design, at the end 4 reported use)
**Fals-Stewart1993**[38]	66	2	therapy	BT	PsyPLA	-	-	12	30·5	55	YBOCS	No	-	Unclear
**Foa2005** [39]	149	4	combination	BT	CLO	BT+ CLO	PLA	12	34·8	48	YBOCS	No	No	-
**Freeman1994** [40]	66	2	drug	FLV	CLO	-	-	10	33·01	47	YBOCS	No	Yes	-
**Freeston1997**[41]	29	2	therapy	CBT	WL	-	-	16	35·8	45	YBOCS	Yes	-	Yes (34% total)
**Goodman1989**[42]	46	2	drug	PLA	FLV	-	-	6	37	55	YBOCS	Yes	No	-
**Goodman1996**[43]	160	2	drug	PLA	FLV	-	-	10	36·65	50	YBOCS	No	Yes	-
**Greist1995**[44]	325	2	drug	SER	PLA	-	-	12	38·64	52	YBOCS	No	Yes	-
**Greist2002**[45]	144	2	therapy	BT	PsyPLA	-	-	10	39	42	YBOCS	Yes	-	Yes (49% total)
**GSK118**[46]	241	3	drug	PLA	PAR	CLO	-	12	37·95	39	YBOCS	No	Yes	-

**GSK526**[47]	146	2	drug	PAR	CLO	-	-	10	30·85	62	YBOCS	No	Yes	-
Hohagen1998[48]	60	2	combination	BT+PLA	BT+FLV	-	-	10	35·5	59	YBOCS	Yes	No	-
**Hollander2003**[49]	253	2	drug	PLA	FLV	-	-	12	37·4	64	YBOCS	No	Yes	-
**Hollander2003b**[50]	348	4	drug	PLA	PAR-20	PAR-40	PAR-60	12	41·36	26	YBOCS	No	Yes	-
**Jaurrieta2008**[51]	38	2	therapy	CBT	WL	-	-	20	31·6	40·4	YBOCS	Yes	-	Yes (100% total)
**Jenike1997**[52]	44	2	drug	PLA	FLX	-	-	10	34·86	48	YBOCS	No	No	-
**Jenike1990a**[53]	19	2	drug	SER	PLA	-	-	10	39·7	21	YBOCS	No	Yes	-
**Jenike1990b**[54]	40	2	drug	PLA	FLV	-	-	10	35·9	47	YBOCS	No	Yes	-
Jones1998[55]	23	2	therapy	CT	WL	-	-	8	38·52	90	Other	Unclear	-	Yes (24% total)
**Kamijima2004**[56]	191	2	drug	PLA	PAR	-	-	12	37·8	62	YBOCS	Yes	Unclear	-
**Khodarahimi2009**[57]	40	2	therapy	BT	WL	-	-	6	24·6	0	YBOCS	No	-	No
**Kobak2005**[58]	60	2	drug	PLA	HYP	-	-	12	37·72	-	YBOCS	No	No	-
**Koran1996**[59]	79	2	drug	FLV	CLO	-	-	10	-	45	YBOCS	No	Yes	-
**Kronig1999**[60]	167	2	drug	SER	PLA	-	-	12	36·76	45	YBOCS	No	Yes	-
**Lindsay1997**[61]	18	2	therapy	BT	PsyPLA	-	-	3	32·8	66	YBOCS	Yes	-	Yes (28% total)
**Lopez-Ibor1996**[62]	55	2	drug	FLX	CLO	-	-	8	34	62	YBOCS	No	Yes	-
Mavissakalian1985[63]	16	2	drug	CLO	PLA	-	-	12	35·9	56	Other	Yes	Yes	-
**McLean2001**[64]	93	2	therapy	CT	BT	-	-	12	35	48	YBOCS	Yes	-	Yes (48% total)
**Milanfranchi1997**[65]	26	2	drug	CLO	FLV	-	-	9	27·35	42	YBOCS	No	Yes	-
**Montgomery1993**[66]	217	4	drug	PLA	FLX-20	FLX-40	FLX-60	8	37·16	47	YBOCS	Yes	Yes	-
**Montgomery2001**[67]	401	4	drug	PLA	CIT-20	CIT-40	CIT-60	12	37·82	54	YBOCS	Yes	Yes	-
**Mundo1997**[68]	30	3	drug	FLV	PAR	CIT	-	10	30·78	30	YBOCS	No	No	-
**Mundo2001**[69]	227	2	drug	CLO	FLV	-	-	10	35·2	45	YBOCS	No	Yes	-
**Nakajima1996**[70]	94	2	drug	FLV	PLA	-	-	8	34·38	46	YBOCS	Unclear	Unclear	-
**Nakatani2005**[71]	31	3	combination	FLV	PsyPLA	BT		12	33·66	68	YBOCS	No	No	-
**O'Connor1999**[72]	29	4	combination	SRI	WL	CBT	CBT+ SRI	20	35·94	37	YBOCS	Yes	No	-
**O'Connor2006**[73]	21	2	drug	PLA	FLV	-	-	20	35·76	57	YBOCS	No	No	-
Perse1987[74]	20	2	drug	PLA	FLV	-	-	8	-	-	Other	Yes	Yes	-
**Shareh2010**[75]	21	3	combination	FLV	CBT	FLV+CBT	-	10	26·84	53	YBOCS	No	No	-
**Sousa2006**[76]	56	2	combination	SER	CBT			12	38·5	77	YBOCS	No	No	-
**Stein2007**[77]	466	4	drug	PLA	PAR	ESCIT-10	ESCIT-20	12	37·75	57	YBOCS	No	Yes	-
Thoren1980[78]	16	2	drug	CLO	PLA	-	-	5	38·9	94	Other	Yes	No	-
**Tollefson1994**[79]	355	4	drug	PLA	FLX-20	FLX-40	FLX-60	13	36·9	55·2	YBOCS	Yes	Yes	-

**Van Oppen1995**[80]	71	2	therapy	CT	BT	-	-	16	34·71	53	YBOCS	Yes	-	No
Volavka1985[81]	23	2	drug	CLO	IMI	-	-	12	29·94	52	YBOCS	No	Yes	-
**Whittal2005**[82]	83	2	therapy	CT	BT	-	-	12	34·89	62·5	YBOCS	Yes	-	Yes (68% total)
**Whittal2010**[83]	73	2	therapy	CT	PsyPLA	-	-	12	31·5	46·6	YBOCS	Yes	-	Yes (52% total)
**Zohar1996**[84]	406	3	drug	PLA	PAR	CLO	-	12	37·94	52	YBOCS	No	Yes	-

BT=Behavioral therapy. CBT=Cognitive-behavioral therapy. CT=Cognitive therapy. CIT=citalopram. CLO=clomipramine. ESCIT= escitalopram. FLV=fluvoxamine. FLX=fluoxetine. HYP=hypericum. N=number. PAR=paroxetine. PLA=placebo. PSYPLA=psychological placebo. SER=sertraline. SRI=serotonin reuptake inhibitor. VEN=venlafaxine; WL=waitlist. YBOCS=Yale-Brown obsessive-compulsive scale. * For studies with at least one drug arm (n=46); ** For studies with psychotherapy arms only (n=18);

Table A2 Raw data used for the YBOCS analysis sorted by study id and number of arms

#study	t[,1]	y[,1]	n[,1]	sd[,1]	t[,2]	y[,2]	n[,2]	sd[,2]	t[,3]	y[,3]	n[,3]	sd[,3]	t[,4]	y[,4]	n[,4]	sd[,4]	Arms
Albert2001[22]	8	18·36	25	7·11	9	17·3	40	6·15	NA	NA	NA	NA	NA	NA	NA	NA	2
Anderson2007[24]	2	23·5	14	6·4	11	16·7	17	6·8	NA	NA	NA	NA	NA	NA	NA	NA	2
Andersson2012[25]	11	12·94	49	6·26	17	18·88	51	4·18	NA	NA	NA	NA	NA	NA	NA	NA	2
Belloch2008[26]	10	8·31	13	8·75	12	6·8	16	3·55	NA	NA	NA	NA	NA	NA	NA	NA	2
Belotto-Silva2012[27]	3	20·29	88	8·05	11	19·97	70	8·48	NA	NA	NA	NA	NA	NA	NA	NA	2
Bergeron2002[28]	3	-9·7	72	7·7	6	-9·6	76	7·9	NA	NA	NA	NA	NA	NA	NA	NA	2
Bisserbe1997[29]	6	-14·3	86	NA	9	-11·71	81	NA	NA	NA	NA	NA	NA	NA	NA	NA	2
Chouinard1990[30]	1	-1·48	44	NA	6	-3·79	43	NA	NA	NA	NA	NA	NA	NA	NA	NA	2
CCSG11991[31]	1	25·11	108	6·34	9	16·23	102	7·37	NA	NA	NA	NA	NA	NA	NA	NA	2
CCSG21991[31]	1	25·59	119	5·78	9	14·7	120	7·45	NA	NA	NA	NA	NA	NA	NA	NA	2
Cordioli2003[32]	2	23·2	24	5·5	11	15·1	23	7·8	NA	NA	NA	NA	NA	NA	NA	NA	2
Cottraux2001[34]	10	-12·1	30	7·8	12	-12·5	30	8·2	NA	NA	NA	NA	NA	NA	NA	NA	2
Denys2003[35]	5	-7·8	72	5·4	8	-7·2	73	7·5	NA	NA	NA	NA	NA	NA	NA	NA	2
Fals-Stewart1993[38]	10	-8·1	31	NA	17	-1·8	32	NA	NA	NA	NA	NA	NA	NA	NA	NA	2
Freeman1994[40]	4	-8·6	28	NA	9	-7·8	19	NA	NA	NA	NA	NA	NA	NA	NA	NA	2
Freeston1997[41]	2	22	14	6	11	12·2	15	9·6	NA	NA	NA	NA	NA	NA	NA	NA	2
Goodman1989[43]	1	28	21	7	4	19·4	21	7	NA	NA	NA	NA	NA	NA	NA	NA	2
Goodman1996[42]	1	-1·71	78	4·88	4	-3·95	78	6·28	NA	NA	NA	NA	NA	NA	NA	NA	2
Greist1995[44]	1	-3·41	84	6·19	6	-5·57	240	6·19	NA	NA	NA	NA	NA	NA	NA	NA	2
Greist2002[45]	10	17·6	55	6·2	17	24·1	66	6·7	NA	NA	NA	NA	NA	NA	NA	NA	2
GSK526[46]	5	-14·26	72	6·33	9	-13·19	69	6·48	NA	NA	NA	NA	NA	NA	NA	NA	2
Hollander2003[49]	1	-5·6	120	7·67	4	-8·5	117	7·57	NA	NA	NA	NA	NA	NA	NA	NA	2
Jaurrieta2008[51]	2	24·6	19	8·9	11	17·8	19	8·4	NA	NA	NA	NA	NA	NA	NA	NA	2
Jenike1997[52]	1	18·7	18	6·1	3	16·2	19	6·3	NA	NA	NA	NA	NA	NA	NA	NA	2
Jenike1990a[53]	1	22·3	9	7·8	6	20·6	10	9·2	NA	NA	NA	NA	NA	NA	NA	NA	2
Jenike1990b[54]	1	21·8	20	7·6	4	18·8	18	4	NA	NA	NA	NA	NA	NA	NA	NA	2
Kamijima2004[56]	1	20·3	94	7·38	5	15·8	94	8·09	NA	NA	NA	NA	NA	NA	NA	NA	2
Khodarahimi2009[57]	2	36·45	20	2·24	10	5·58	20	2·39	NA	NA	NA	NA	NA	NA	NA	NA	2
Kobak2005[58]	1	19·87	30	7·46	13	19·75	30	7·46	NA	NA	NA	NA	NA	NA	NA	NA	2

Koran1996 [59]	4	17·8	34	7·7	9	17	39	8·55	NA	NA	NA	NA	NA	NA	NA	NA	2
Kronig1999 [60]	1	-4·14	79	NA	6	-8·5	85	NA	NA	NA	NA	NA	NA	NA	NA	NA	2
Lindsay1997 [61]	10	11	9	3·81	17	25·89	9	5·8	NA	NA	NA	NA	NA	NA	NA	NA	2
Lopez-Ibor1996 [62]	3	-7·5	30	9·29	9	-8·9	24	7·13	NA	NA	NA	NA	NA	NA	NA	NA	2
McLean2001 [64]	12	16·1	31	6·7	10	13·2	32	7·2	NA	NA	NA	NA	NA	NA	NA	NA	2
Milanfranchi1997 [65]	4	18·4	13	9·2	9	16·5	12	11	NA	NA	NA	NA	NA	NA	NA	NA	2
Nakajima1996 [70]	1	-1·9	33	7·2	4	-7·1	60	7·03	NA	NA	NA	NA	NA	NA	NA	NA	2
O'Connor1999 [72]	2	17·5	6	4	11	13·3	6	8·6	NA	NA	NA	NA	NA	NA	NA	NA	2
O'Connor2006 [73]	1	25·4	10	3·5	4	24	11	4·7	NA	NA	NA	NA	NA	NA	NA	NA	2
Sousa2006 [76]	6	-7·36	25	NA	11	-10·8	25	NA	NA	NA	NA	NA	NA	NA	NA	NA	2
Tollefson1994 [79]	1	-0·8	89	5·66	3	-5·44	266	7·88	NA	NA	NA	NA	NA	NA	NA	NA	2
Van Oppen1995 [80]	10	17·9	29	9	12	13·4	28	9·4	NA	NA	NA	NA	NA	NA	NA	NA	2
Whittal2005 [82]	10	10·41	29	7·6	12	10·6	30	7·1	NA	NA	NA	NA	NA	NA	NA	NA	2
Whittal2010 [83]	12	6·43	37	4·77	17	9·1	30	6·48	NA	NA	NA	NA	NA	NA	NA	NA	2
Mundo2001 [69]	4	-12·2	115	NA	9	-12	112	NA	NA	NA	NA	NA	NA	NA	NA	NA	2
GSK118 [47]	1	-4·61	75	7·53	5	-5·61	79	7·47	9	-7·73	78	7·42	NA	NA	NA	NA	3
Mundo1997 [68]	4	16·2	10	8·9	5	21·6	9	7·6	7	19·8	11	10·1	NA	NA	NA	NA	3
Nakatani2005 [71]	4	20·2	10	9·4	10	12·9	10	4·9	17	28·4	8	5·5	NA	NA	NA	NA	3
Shareh2010 [75]	4	16·66	6	3·2	11	7	7	2·38	14	8·5	6	2·42	NA	NA	NA	NA	3
Zohar1996 [84]	1	-4·2	99	7·2	5	-6·4	201	7·1	9	-7	99	6·8	NA	NA	NA	NA	3
Foa2005 [39]	1	22·2	26	6·4	9	18·2	36	7·8	10	11	29	7·9	15	10·5	31	8·2	4
Hollander2003b [49]	1	-3·33	89	NA	5	-4·14	88	NA	5	-6·35	86	NA	5	-7·34	85	NA	4
Montgomery1993 [67]	1	-3·7	56	5·98	3	-5·13	52	6·41	3	-4·76	52	6·89	3	-6·07	54	6·92	4
Montgomery2001 [66]	1	-5·6	101	6·9	7	-8·4	102	7·3	7	-8·9	98	7	7	-10·4	100	6·9	4
Stein2007 [77]	1	-8·46	113	8·08	5	-11·67	116	8·40	16	-11·43	112	8·25	16	-12·14	114	8·22	4

t[i]: type of treatment per arm [i] (1=placebo, 2=waitlist,3=fluoxetine, 4=fluvoxamine, 5=paroxetine, 6=sertraline, 7=citalopram, 8=venlafaxine, 9=clomipramine, 10=behavioural therapy, 11=CBT, 12=cognitive therapy, 13=Hypericum, 14=fluvoxamine and CBT, 15= clomipramine and behavioural therapy, 16=escitalopram, 17=psychological placebo; y[i]:total mean YBOCS scores at end of study (positive) or mean change from baseline (negative); n[i]: total number of patients in arm [i]; sd[i]: standard deviation of mean total score or change from baseline [i]. NA: not applicable.

Table A3 List of excluded Studies

Reference Title		Reason for Exclusion
1.	Aigner M, Demal U, Zitterl W et al. Behavioural group therapy for obsessive-compulsive disorder. Verhaltenstherapie 2004; 14:7-14.	controlled but not randomised
2.	Akouchekian S, Jamshidian Z Maracy MR et al. Religious cognitive behavioral therapy in religious oriented obsessive compulsive disorder. European Psychiatry [abstracts from the 19th European Congress of Psychiatry, EPA 2011 Mar 12-15; Vienna, Austria]	duplicate - early congress abstract of the full paper
3.	Akuchekian S, Jamshidian Z, Maracy M et al. Effectiveness of religious-cognitive-behavioral therapy on religious oriented obsessive compulsive disorder and its co-morbidity. Journal of Isfahan Medical School 2011; 28:1	special subgroup of OCD patients with religious-oriented symptoms (in Arab)
4.	Askin R, Turan M, Çilli AS et al. Clomipramine versus Sertraline in the Treatment of Obsessive Compulsive Disorder. Bull Clin Psychopharmacol 1999; 9:133-138	Usable data only for dichotomous outcome. No variability measure for continuous outcome
5.	Balkom AV, Haan ED, Oppen PV et al. Cognitive-Behavioral Therapy Versus the Combination with Fluvoxamine in the Treatment of OCD. 150th Annual Meeting of the American Psychiatric Association. San Diego, California, USA. 17-22 May, 1997.	duplicate reporting- Early congress abstract of the vanBalkom1998 paper
6.	Belloch A, Cabedo E, Carrio C et al. Group versus individual cognitive treatment for Obsessive-Compulsive Disorder: Changes in non-OCD symptoms and cognitions at post-treatment and one-year follow-up. Psychiatry Res 2011; 187:174-9	secondary analysis of Cabedo2010 paper which has also been excluded
7.	Black D.W, Monahan P., Gable J et al. Hoarding and treatment response in 38 nondepressed subjects with obsessive-compulsive disorder. J Clin Psychiatry 1998; 59:420-5	duplicate data, PAR vs PLA already included in Hollander2003. CBT arm not randomised
8.	Cabedo E, Belloch A, Carrio C et al. Group versus individual cognitive treatment for obsessive-compulsive disorder:	control intervention not covered (comparison between different

	Changes in severity at post-treatment and one-year follow-up. Behav Cogn Psychother 2010; 38:227-32	forms of the same therapy)
9.	Denys D, van Megen HJ, van der Wee N et al. A double-blind switch study of paroxetine and venlafaxine in obsessive-compulsive disorder. J Clin Psychiatry 2004; 65:37-43	extension of the Dennys2003 study in non responders (treatment refractory population)
10.	Dougherty DD, Jameson M, Deckersbach T et al. Open-label study of high (30 mg) and moderate (20 mg) dose escitalopram for the treatment of obsessive-compulsive disorder. Int Clin Psychopharmacol 2009; 24:306-11	dose ranging study of the same drug - no comparator
11.	Eli, L. Fluoxetine Treatment for Obsessive Compulsive Disorder in Children and Adolescents [Trial 3032]	duplicate with Geller
12.	Fineberg NA, Hughes A, Gale TM et al. Group cognitive behaviour therapy in obsessive-compulsive disorder (OCD): A controlled study. Int J Psychiatry Clin Pract 2005; 9:257-63	this paper used systematic and not random sampling
13.	Franklin ME, Abramowitz JS et al. Cognitive-behavioral therapy with and without medication in the treatment of obsessive-compulsive disorder. Professional Psychology: Research and Practice 2002; 33:162–168	observational study stratified by medication - not an experimental study
14.	Giasuddini NA, Nahar JS, Morshed NM et al. Efficacy of combination of fluoxetine and cognitive behavioral therapy and fluoxetine alone for the treatment of obsessive compulsive disorder. Pak J Pharm Sci 2013; 26:95-8	uncertain if truly randomised (abstract does not mention randomised, baseline scores of the scale used almost marginally significantly different between the two groups with p=0.07)), unable to find if the symptom scale used (DUOCS) has been validated or not - reference given unable to locate, this scale has not been used again in research
15.	GlaxoSmithKline 1993a	early report of the Hollander2003 data set
16.	GlaxoSmithKline 1993b	duplicate with Zohar1996
17.	Goodman WK, Lydiard RB, Rubin A, et al. Safety of sertraline in long-term OCD treatment: preliminary results of a multicenter study. 152nd Annual Meeting of the American Psychiatric Association. 1999 May 15-20, Washington DC	relapse prevention study
18.	Goodman WK, Price LH, Delgado PL, et al. Specificity of serotonin reuptake inhibitors in the treatment of obsessive-	see control intervention

	compulsive disorder Comparison of fluvoxamine and desipramine. Arch Gen Psychiatry 1990; 47:577-85	
19.	Greist, JH. Fluvoxamine in obsessive compulsive disorder: A multicenter parallel design double-blind placebo-controlled trial. Clin Neuropharm 1992; 15 (Suppl. 1, Pt. B): 310B	abstract report with no data given. This is one of the two pivotal studies of fluvoxamine from Solvay but only the second - Goodman1996 - has been published. Greist1995 in a meta-analysis has combined the two trials but no data can be used either
20.	Greist J H, Jefferson J W, Rosenfeld R et al. A 1 year double-blind placebo-controlled fixed dose study of sertraline in the treatment of obsessive-compulsive disorder. Int Clin Psychopharmacol 1995; 10:57-65	duplicate with CCSG1991
21.	Hewlett, WA, Vinogradov S and Agras WS. Clomipramine, clonazepam, and clonidine treatment of obsessive-compulsive disorder. J Clin Psychopharmacol 1992; 12:420-30	comparator not covered (clonazepam, clonidine)
22.	Hoehn-Saric R, Ninan P, Black DW et al. Multicenter double-blind comparison of sertraline and desipramine for concurrent obsessive-compulsive and major depressive disorders. Arch Gen Psychiatry 2000; 57:76-82	see diagnosis and control not covered
23.	Hohagen F, König A, Rasche-Räuchle H, et al. Behavior therapy and fluvoxamine versus behavior therapy and placebo: Results of a multicenter study	duplicate - early congress abstract of the Hohagen1998 paper
24.	Holland R, Vardy A and Bolt G. A comparison of fluvoxamine (FL) and clomipramine (CLO) in the treatment of obsessive compulsive disorder (OCD)	abstract congress about tolerability including previously published data from solvay
25.	Insel TR, Murphy DL, Cohen R. M et al. Obsessive-compulsive disorder. A double-blind trial of clomipramine and clorgyline. Arch Gen Psychiatry 1983; 40:605-12	control intervention not covered
26.	Jakubovski E, Diniz JB, Valerio C et al. Clinical predictors of long-term outcome in obsessive-compulsive disorder. Depress Anxiety 2013; 30:763-72	duplicate with belotto - secondary
27.	Jenike MA, Baer L, Summergrad P et al. Obsessive-compulsive disorder: a double-blind, placebo-controlled trial of	duplicate with CCSG1991

	clomipramine in 27 patients. Am J Psychiatry 1989; 146:1328-30	
28.	Jonsson H, Hougaard E and Bennedsen BE. Randomized comparative study of group versus individual cognitive behavioural therapy for obsessive compulsive disorder. Acta Psychiatr Scand 2011; 123:387-97	control intervention not covered (comparison between different forms of the same therapy)
29.	Karabanow O. Double-blind controlled study in phobias and obsessions. J Int Med Res 1977; Suppl 5:42-8	not exclusively OCD (phobias) - unstardised diagnosis
30.	Kearns C, Tone Y, Rush G et al. Effectiveness of group-based cognitive-behavioural therapy in patients with obsessive-compulsive disorder. The Psychiatrist 2010; 34:6-9	uncontrolled case series (not randomised)
31.	Khan MN, Hotiana UA and Ahmad S. Escitalopram in the treatment of obsessive-compulsive disorder: a double blind placebo control trial. J Ayub Med Coll Abbottabad 2007; 19:58-63	first phase of the study open label uncontrolled trial, 2nd phase randomised responders only for relapse prevention
32.	Koran LM, Cain JW, Dominguez RA et al. Are fluoxetine plasma levels related to outcome in obsessive-compulsive disorder? Am J Psychiatry 1996; 153:1450-4	duplicate with Tolefson
33.	Kudo Y. A placebo controlled double blind study in obsessive compulsive disorder with fluvoxamine	early congress report of the Nakajima paper
34.	Leonard HL, Swedo SE, Rapoport JL, et al. Treatment of obsessive-compulsive disorder with clomipramine and desipramine in children and adolescents A double-blind crossover comparison. Arch Gen Psychiatry 1989; 46:1088-92	comparator not covered
35.	Ma JD, Wang CH, Li HF, et al. Cognitive-coping therapy for obsessive-compulsive disorder: a randomized controlled trial J Psychiatr Res 2013; 47:1785-90	non extractable data for non-resistant patients: this study has included patients that were treatment resistant within the study but no separate data are given for those non-resistant
36.	Mallya GK, White K, Waternaux C and Quay S. Short and long term treatment of obsessive compulsive disorder with fluvoxamine. Annals of Clinical Psychiatry 1992; 4:77-80	duplicate data also reported in Goodman1996
37.	Marks IM, Lelliott P, Basoglu M, et al. Clomipramine, self-exposure and therapist-aided exposure for obsessive-compulsive rituals. Br J Psychiatry 1988; 152:522-34.	No extractable data for treatment comparisons

38.	Marks IM, Stern RS, Mawson D et al. Clomipramine and exposure for obsessive-compulsive rituals. Br J Psychiatry 1980; 136:1-25.	No extractable data for treatment comparisons- OCD diagnosis not standardised
39.	Mavissakalian MR, Jones B, Olson S et al. Clomipramine in obsessive-compulsive disorder: clinical response and plasma levels. J Clin Psychopharmacol 1990; 10:261-8.	duplicate with CCSG1991
40.	Montgomery SA. Clomipramine in obsessional neurosis: a placebo-controlled trial. Pharmacological Medicine 1980; 1:189–192	duplicate - crossover data at the point of cross-over, also reported later in Montgomery1990
41.	Mundo E, Bianchi L and Bellodi L. Efficacy of fluvoxamine, paroxetine, and citalopram in the treatment of obsessive-compulsive disorder: a single-blind study. J Clin Psychopharmacol 1997; 17:267-71	dose ranging study of the same drug - no comparator
42.	Mundo E, Maina G. and Uslenghi C. Multicentre, double-blind, comparison of fluvoxamine and clomipramine in the treatment of obsessive-compulsive disorder. Int Clin Psychopharmacol 2000; 15:69-76.	early report of a subset of the data that also reported in Mundo2001 (14 out of the 40 centres)
43.	Muroff J, Steketee G, Bratiotis C et al. Group cognitive and behavioral therapy and bibliotherapy for hoarding: A pilot trial. Depress Anxiety 2012; 29:597-604.	hoarding disorder not OCD
44.	Nazari H, Momeni N, Jariani M et al. Comparison of eye movement desensitization and reprocessing with citalopram in treatment of obsessivecompulsive disorder. Int J Psychiatry Clin Pract 2011; 15:270-4	main intervention not covered - eye movement desensitization and reprocessing (EMDR)
45.	Olatunji BO, Rosenfield D, Tart CD et al. Behavioral versus cognitive treatment of obsessive-compulsive disorder: an examination of outcome and mediators of change. J Consult Clin Psychol 2013; 81:415-28.	reports same data with Cottraux2001 but different method of analysis and treating missing data (multilevel instead LOCF)
46.	Omranifard V, Akuchakian S, Almasi A et al. Effect of religious cognitive-behavior therapy on religious content obsessive compulsive disorder and marital satisfaction [conference abstract]	duplicate with the Akuchakian2011 paper that has been excluded
47.	Pigott TA, L'Heureux F, Rubenstein CS et al. A double-blind, placebo controlled study of trazodone in patients with obsessive-compulsive disorder. J Clin Psychopharmacol 1992; 12: 156-62.	Intervention not included (trazodone)
48.	Pigott TA, Pato MT, Bernstein SE et al. Controlled comparisons of clomipramine and fluoxetine in the treatment of	data not extractable at the point of cross-over

	obsessive-compulsive disorder. Behavioral and biological results. Arch Gen Psychiatry 1990; 47:926-32	
49.	Rapoport, J, Elkins R and Mikkelsen E. Clinical controlled trial of chlorimipramine in adolescents with obsessive-compulsive disorder. Psychopharmacol Bull 1980; 16:61-3	duplicate data - this is an early report of the Flament1985 study (this is reported and cited in the later Flament study
50.	Richter P, Witheridge K, Daskalakis ZJ et al. Investigation of predictors of drug response in obsessive compulsive disorder (OCD) [conference abstract]	congress abstract only - crossover of escitalopran vs clomipramine but no usable data given
51.	Rouillon F. A double-blind comparison of fluvoxamine and clomipramine in OCD	duplicate, early congress report of the Mundo2001 study
52.	Shareh H, Gharaie B and Vahid MKA. Comparison between metacognitive therapy, fluvoxamine and combined therapy in the improvement of thought control strategies and stop signal criteria in obsessive compulsive disorder. [Farsi (Iranian)]. IJPCP 2011; 17:199-207	duplicate publication of Shareh2011a
53.	Shareh H, Gharraee B and Vahid MKA. Comparison of metacognitive therapy, fluvoxamine and combined treatment in Improving metacognitive beliefs and subjective distress of patients with obsessive-compulsive disorder. [Farsi (Iranian)]. Advances in Cognitive Science 2011; 12:45-59	secondary analysis of Sharreh2010
54.	Shavitt R, Valerio C, Diniz, JB et al. Clinical predictors of treatment outcome in obsessive-compulsive disorder: A two-year follow-up [conference abstract]	congress abstract - extension of the Belotto-Silava2012 dataset
55.	Sibon I, Leyton M, Gravel P et al. CBT vs sertraline in OCD: Effects on brain regional serotonin synthesis index ACNP, Dec. 11-15, 2005, Waikoloa, HI	congress abstract, data not given for analysis, unable to decide on inclusion criteria
56.	Solyom L and Sookman D. A comparison of clomipramine hydrochloride (Anafranil) and behaviour therapy in the treatment of obsessive neurosis. J Int Med Res 1977; Suppl 5:49-61	not randomised
57.	Stein DJ, Hollander E, Mullen LS, et al. Comparison of clomipramine, alprazolam and placebo in the treatment of obsessive compulsive disorder. Human Psychopharmacology: Clinical and Experimental 1992; 7:389-395	continuous data not extractable for treartment comparisons
58.	Stein DJ, Tonnior B. and Andersen EW. Escitalopram in the treatment of ocd	early congress abstract of the full Stein2007 paper

59.	Steketee G, Frost RO, Tolin DF et al. Waitlist-controlled trial of cognitive behavior therapy for hoarding disorder. Depress Anxiety 2010; 27:476-84	hoarding disorder not OCD
60.	Tamimi Raed R, Mavissakalian MR, Jones B et al. Clomipramine versus fluvoxamine in obsessive-compulsive disorder Annals of Clinical Psychiatry 1991; 3:275-279	unblinded (open label) controlled trial
61.	Turner SM, Jacob RG, Beidel DC and Himmelhoch J. Fluoxetine treatment of obsessive-compulsive disorder. J Clin Psychopharmacol 1985; 5:207-12	uncontrolled study, only one fluoxetine arm
62.	Vallejo J, Olivares J, Marcos T et al. Clomipramine versus phenelzine in obsessive-compulsive disorder. A controlled clinical trial. Br J Psychiatry 1992; 161:665-70	comparator (phenelzine) - not covered
63.	van Balkom AJ, de Haan E, van Oppen P et al. Cognitive and behavioral therapies alone versus in combination with fluvoxamine in the treatment of obsessive compulsive disorder. J Nerv Ment Dis 1998; 186:492-9	the usable comparison (CBT vs BT for 16 weeks has been fully reported in vanOppen1995 study, therefore this reports is duplicate. Remaining arms cannot beused due to the complexity of the design)
64.	Wheadon DE, Bushnell WD and Steiner MA. A fixed-dose comparison of 20, 40 or 60 mg paroxetine to placebo in the treatment of obsessive-compulsive disorder. Paper presented at the annual meeting of the American College of Neuropsychopharmacology Honolulu 1993	early report of the Hollander2003 data set
65.	Wilhelm S, Steketee G, Fama JM et al. Modular cognitive therapy for obsessive-compulsive disorder: A wait-list controlled trial. J Cogn Psychother 2009; 23:294-305	not random assignment (but "according to therapist availability)
66.	Williams TI, Salkovskis P, White H et al. Trialling cognitive behaviour therapy for children with OCD: a randomised controlled trial	duplicate - early congress abstract of the Williams2010 full paper
67.	Wootton BM, Dear BF, Johnston L et al. Remote treatment of obsessive-compulsive disorder: A randomized controlled trial. Journal of Obsessive-Compulsive and Related Disorders 2013; 2:375-384	main aim of the paper to compare different form of same treatment

68.	Yaryura-Tobias JA and Neziroglu FA. Venlafaxine in obsessive-compulsive disorder. Arch Gen Psychiatry 1996; 53:653-4	this is a letter from a small pilot study that according to the authors is double blind but the results have not been published. The authors report very general trends and the study is negative. It is not possible to extraxt any information
69.	Alaghband-Rad J, Hakimshooshtary M. A randomized controlled clinical trial of citalopram versus fluoxetine in children and adolescents with obsessive-compulsive disorder (OCD). Eur Child Adolesc Psychiatry 2009 Mar;18(3):131-5.	Included in Children & Adolescent subset
70.	Asbahr FR, Castillo AR, Ito LM, Latorre MR, Moreira MN, Lotufo-Neto F. Group cognitive-behavioral therapy versus sertraline for the treatment of children and adolescents with obsessive-compulsive disorder. J Am Acad Child Adolesc Psychiatry 2005; 44:1128-1136.	Included in Children & Adolescent subset
71.	Barrett P, Healy-Farrell L, March JS. Cognitive-behavioral family treatment of childhood obsessive-compulsive disorder: a controlled trial. J Am Acad Child Adolesc Psychiatry 2004; 43:46-62.	Included in Children & Adolescent subset
72.	Bolton D, Perrin S. Evaluation of exposure with response-prevention for obsessive compulsive disorder in childhood and adolescence. J Behav Ther Exp Psychiatry 2008; 39:11-22.	Included in Children & Adolescent subset
73.	Bolton D, Williams T, Perrin S, Atkinson L, Gallop C, Waite P, Salkovskis P. Randomized controlled trial of full and brief cognitive-behaviour therapy and wait-list for paediatric obsessive-compulsive disorder. J Child Psychol Psychiatry 2011; 52:1269-1278.	Included in Children & Adolescent subset
74.	DeVeaugh-Geiss J, Moroz G, Biederman J, et al. Clomipramine hydrochloride in childhood and adolescent obsessive-compulsive disorder: a multicenter trial. J Am Acad Child Adolesc Psychiatry 1992; 31:45-49.	Included in Children & Adolescent subset
75.	Flament MF, Rapoport JL, Berg CJ, Sceery W, Kilts C, Mellström B, et al. Clomipramine treatment of childhood obsessive–compulsive disorder: a double-blind controlled study. Arch Gen Psychiatry 1985;42:977–83.	Included in Children & Adolescent subset
76.	Freeman JB, Garcia AM, Coyne L, Ale C, Przeworski A, Himle M, Compton S, Leonard HL. Early childhood OCD:	Included in Children & Adolescent subset

	preliminary findings from a family-based cognitive-behavioral approach. J Am Acad Child Adolesc Psychiatry 2008; 47:593-602.	
77.	Geller DA, Hoog SL, Heiligenstein JH, et al. Fluoxetine treatment for obsessive-compulsive disorder in children and adolescents: a placebo-controlled clinical trial. J Am Acad Child Adolesc Psychiatry 2001; 40:773-779.	Included in Children & Adolescent subset
78.	GSK. A Randomized, Multicenter, 10-Week, Double-Blind, Placebo- Controlled, Flexible-Dose Study to Evaluate the Efficacy and Safety of Paroxetine in Children and Adolescents with Obsessive-Compulsive Disorder (OCD) (29060/704).	Included in Children & Adolescent subset
79.	De Haan E, Hoogduin KA, Buitelaar JK, et al. Behavior therapy versus clomipramine for the treatment of obsessive-compulsive disorder in children and adolescents. J Am Acad Child Adolesc Psychiatry 1998; 37:1022-1029.	Included in Children & Adolescent subset
80.	Liebowitz MR, Turner SM, Piacentini J, et al. Fluoxetine in children and adolescents with OCD: a placebo-controlled trial. J Am Acad Child Adolesc Psychiatry 2002; 41:1431-1438.	Included in Children & Adolescent subset
81.	March JS, Johnston H, Jefferson JW, et al. Do subtle neurological impairments predict treatment resistance to clomipramine in children and adolescents with obsessive-compulsive disorder? J Child Adolesc Psychopharmacol 1990; 1:133-140.	Included in Children & Adolescent subset
82.	March JS, Biederman J, Wolkow R, et al. Sertraline in children and adolescents with obsessive-compulsive disorder: a multicenter randomized controlled trial. JAMA 1998; 280:1752-1756.	Included in Children & Adolescent subset
83.	Neziroglu F, Yaryura-Tobias JA, Walz J, et al. The effect of fluvoxamine and behavior therapy on children and adolescents with obsessive-compulsive disorder. J Child Adolesc Psychopharmacol 2000; 10:295-306.	Included in Children & Adolescent subset
84.	Piacentini J, Bergman RL, Chang S, Langley A, Peris T, Wood JJ, McCracken J. Controlled comparison of family cognitive behavioral therapy and psychoeducation/relaxation training for child obsessive-compulsive disorder. J Am Acad Child Adolesc Psychiatry 2011; 50:1149-1161.	Included in Children & Adolescent subset

85.	POTS. Cognitive-behavior therapy, sertraline, and their combination for children and adolescents with obsessive-compulsive disorder: the Pediatric OCD Treatment Study (POTS) randomized controlled trial. JAMA 2004; 292:1969-1976.	Included in Children & Adolescent subset
86.	Riddle MA, Scahill L, King RA, et al. Double-blind, crossover trial of fluoxetine and placebo in children and adolescents with obsessive-compulsive disorder. J Am Acad Child Adolesc Psychiatry 1992;31:1062-1069.	Included in Children & Adolescent subset
87.	Riddle MA, Reeve EA, Yaryura-Tobias JA, et al. Fluvoxamine for children and adolescents with obsessive-compulsive disorder: a randomized, controlled, multicenter trial. J Am Acad Child Adolesc Psychiatry 2001;40:222-229.	Included in Children & Adolescent subset
88.	Storch EA, Caporino NE, Morgan JR, Lewin AB, Rojas A, Brauer L, Larson MJ, Murphy TK. Preliminary investigation of web-camera delivered cognitive-behavioral therapy for youth with obsessive-compulsive disorder. Psychiatry Res 2011; 189:407-412.	Included in Children & Adolescent subset
89.	Storch EA, Bussing R, Small BJ, Geffken GR, McNamara JP, Rahman O, Lewin AB, Garvan CS, Goodman WK, Murphy TK. Randomized, placebo-controlled trial of cognitive-behavioral therapy alone or combined with sertraline in the treatment of pediatric obsessive-compulsive disorder. Behav Res Ther 2013; 51:823-829.	Included in Children & Adolescent subset
90.	Williams TI, Salkovskis PM, Forrester L, Turner S, White H, Allsopp MA. A randomised controlled trial of cognitive behavioural treatment for obsessive compulsive disorder in children and adolescents. Eur Child Adolesc Psychiatry 2010; 19:449-456.	Included in Children & Adolescent subset
91.	Mahoney AE, Mackenzie A, Williams AD, Smith J, Andrews G. Internet cognitive behavioural treatment for obsessive compulsive disorder: A randomised controlled trial. Behav Res Ther. 2014;63C:99-106.	Intervention is a specially developed self-help variant of CBT with no therapist contact
92.	Hu XZ, Ma JD, Huang P, Shan XW, Zhang ZH, Zhang JH, Ouyang H, Kou SJ, Li ZR, Wang SF, Zhao HZ, Wang H, Wang CH. Highly efficacious cognitive-coping therapy for overt or covert compulsions. Psychiatry Res. 2015 Oct 30;229(3):732-8.	Intervention is a newly developed adjuvant psychotherapy variant of CT (cognitive-coping therapy)

93.	Goetz AR, Lee HJ. The effects of preventive and restorative safety behaviors on a single-session of exposure therapy for contamination fear. J Behav Ther Exp Psychiatry. 2015;46:151-7.	Tested a specific component of exposure therapy (BT) in a subgroup of patients with contamination fear
94.	Kobak KA, Greist R, Jacobi DM, Levy-Mack H, Greist JH. Computer-assisted cognitive behavior therapy for obsessive-compulsive disorder: a randomized trial on the impact of lay vs. professional coaching. Ann Gen Psychiatry. 2015;14:10.	Tested the effectiveness of a method of delivery (professional vs. lay coaching) of a variant of CBT
95.	McLean CP, Zandberg LJ, Van Meter PE, Carpenter JK, Simpson HB, Foa EB. Exposure and response prevention helps adults with obsessive-compulsive disorder who do not respond to pharmacological augmentation strategies. J Clin Psychiatry. 2015; 76(12):1653-7	Trial of Behavioral therapy in treatment resistant patients as a third step in a stepped care trial

Table A4 Meta-regression of effect modifiers

**Outcome: Mean YBOCS reduction**	
**Included Covariate**	Coefficient (95% CrI)
Publication date (continuous)	0.14 (-0.11 to 0.39)
Trial length (continuous)	0.31 (-0.26 to 0.86)
Comorbid Depression (Binary, 1=yes)	-1.24 (-4.34 to 1.78)
Pharmaceutical Industry sponsorship (Binary, 1=yes)	-0.40 (-4.33 to 3.41)
